# Impacts of spruce budworm defoliation on the habitat of woodland caribou, moose, and their main predators

**DOI:** 10.1002/ece3.8695

**Published:** 2022-03-18

**Authors:** Catherine Chagnon, Mathieu Bouchard, David Pothier

**Affiliations:** ^1^ 4440 Département des sciences du bois et de la forêt Centre d’étude de la forêt Université Laval Québec Quebec Canada

**Keywords:** *Choristoneura fumiferana* (Clemens), insect outbreaks, Québec, *Rangifer tarandus caribou* (Gmelin), spruce budworm, understory, woodland caribou

## Abstract

Forest logging has contributed to the decline of several woodland caribou populations by causing the fragmentation of mature coniferous stands. Such habitat alterations could be worsened by spruce budworm (SBW) outbreaks. Using 6201 vegetation plots from provincial inventories conducted after the last SBW outbreak (1968–1992) in boreal forests of Québec (Canada), we investigated the influence of SBW‐caused tree defoliation and mortality on understory vegetation layers relevant to woodland caribou and its main predators. We found a positive association between severe outbreaks and the cover of most groups of understory plant species, especially in stands that were dominated by balsam fir before the outbreak, where a high canopy openness particularly benefited relatively fast‐growing deciduous plants. Such increases in early successional vegetation could provide high‐quality forage for moose, which is likely to promote higher wolf densities and increase predation pressure on caribou. SBW outbreaks may thus negatively affect woodland caribou by increasing predation risk, the main factor limiting caribou populations in managed forests. For the near future, we recommend updating the criteria used to define critical caribou habitat to consider the potential impacts of spruce budworm defoliation.

## Graphical abstract

Spruce budworm outbreaks affect boreal forest structure and cause an increase in the cover of understory plant species. This could have negative impacts on woodland caribou populations in eastern Canada by increasing the abundance of alternate preys for key caribou predators.

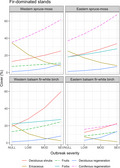

## 1 INTRODUCTION

Since the end of the 19th century, the geographic range and population size of woodland caribou (*Rangifer tarandus caribou* Gmelin) have gradually declined (Courtois et al., 2003) leading to its designation as threatened with extinction in Canada in 2002 (COSEWIC, 2002) and as vulnerable in Québec in 2005 (MFFP, 2005). While the early decline of woodland caribou populations was attributed to overhunting (Bergerud, 1974), the increased predation risk associated with forest logging appears to be the main cause of some recent population reductions across Canada (Wittmer et al., 2005; Wittmer et al., 2007 in British Columbia; Bowman et al., 2010 in Ontario; Courtois et al., 2007 in Québec). Even though caribou could benefit up to a point from the increased abundance of forage (deciduous or coniferous; Table 3), it tends to prioritize predator avoidance (Hins et al., 2009; McGreer et al., 2015) over forage availability when selecting habitat, which generally restrains caribou to less productive environments such as mature forests (Hins et al., 2009). By favoring early successional vegetation, logging provides suitable habitat for moose (*Alces alces* L.), which indirectly increases predation on caribou by supporting higher predator densities, particularly wolf (*Canis lupus* L.; Bowman et al., 2010 in Ontario; Mosnier, Boisjoly, et al., 2008, Courbin et al., 2009, in Québec; James et al., 2004; Peters et al., 2013 in Alberta; Rettie & Messier, 2000 in Saskatchewan). Infrastructures left by logging such as cutlines, trails, and roads also provide forage for moose and facilitate movement by wolf and are thus considered to have a strong negative effect upon caribou habitat (Dickie et al., 2016; Wittische et al., 2021). For these reasons, caribou survival tends to be negatively correlated to the extent of regenerating stands located within its home range (Courtois et al., 2007; Wittmer et al., 2007).

The growing extent of areas affected by forest logging throughout the 20th century has altered disturbance dynamics and increased disturbance frequency within the boreal forest (Boucher & Grondin, 2012; Guindon et al., 2014). In this context, the interaction between logging and natural disturbances could have critical implications for woodland caribou habitat. Although fire is generally understood as the main natural disturbance in the boreal forest (Stralberg et al., 2018), spruce budworm (*Choristoneura fumiferana* Clemens; SBW) outbreaks also represent a major natural disturbance in eastern North America, affecting millions of hectares of forests (Bouchard & Auger, 2014; Sturtevant et al., 2015). Outbreaks occur periodically every 30–40 years (Jardon et al., 2003) and result in the defoliation of its hosts, leading to important tree mortality. Tree mortality levels vary according to stand composition, number of consecutive years with severe defoliation, and stand age (Bouchard et al., 2005). Balsam fir (*Abies balsamea* Mill.) tends to suffer greater SBW defoliation than other hosts (Hennigar et al., 2008) and can be affected by mortality levels reaching up to 90% of individuals (Bouchard et al., 2005).

Tree defoliation and mortality induced by SBW outbreaks causes changes in canopy openness (D’Aoust et al., 2004), which can alter stand dynamics and understory composition (Kneeshaw & Bergeron, 1998; Sánchez‐Pinillos et al., 2019). While balsam fir recruitment tends to be abundant in stands that suffered heavy mortality (Virgin & MacLean, 2017), increased canopy openness also benefits shade‐intolerant deciduous species present in the understory, which may form an important component of the canopy in the decades following the disturbance (D’Aoust et al., 2004; Sánchez‐Pinillos et al., 2019). These shifts in understory structure and composition due to insect pests (Fourrier et al., 2015; Kemball et al., 2005) may be detrimental for animal species that are dependent on mature stands, including woodland caribou.

Spruce budworm outbreaks have historically occurred mainly within the southernmost part of woodland caribou's distributional range, yet a northward shift in SBW outbreak distribution is expected with climate warming (Navarro et al., 2018; Régnière et al., 2012), potentially increasing the overlap between the ranges of the SBW and woodland caribou. Moreover, because caribou habitat has been significantly fragmented by forest logging over the last decades (Fryxell et al., 2020), SBW outbreaks could affect the remaining mature coniferous patches and negatively affect already fragile caribou populations. Still, the potential effect of SBW outbreaks on the habitat of caribou, its main predators, and moose (the main alternate prey of wolf) remains to be investigated. This is of major importance, because the SBW could potentially affect boreal forest understories synchronously across 1000’s of km^2^. In this study, we evaluated the influence of the last SBW outbreak (1968–1992) on the understory of boreal stands in the vicinity of woodland caribou distributional range within the province of Québec. We investigated the effect of SBW defoliation on key understory plant species that are important for either caribou, moose, or their predators, and the potential importance of these changes are interpreted in the context of the SBW outbreak that is currently unfolding in this territory.

## 2 MATERIALS AND METHODS

### 2.1 Plot selection and data compilation

We used a network of 28,425 ecological observation plots (EOPs) that were established between 1986 and 2000 by the Ministère des Forêts, de la Faune et des Parcs (MFFP) of the Government of Québec. This wide‐ranging inventory covered most of the continuous forest of Québec and characterized biological and physical attributes of forest stands that had established in different topographical, geological, and geomorphological contexts. EOPs consisted of 400 m^2^ circular plots organized along transects comprising of between five and seven plots so that one EOP was established every 15 to 25 km^2^ across the whole inventoried area (see Saucier et al., 1994 for more details on EOPs). Vegetation cover (percent cover for every species in the 400 m^2^ plot) in the under‐ and overstory strata was measured in each EOP, and the presence of significant tree mortality (25%–75% and >75% of stand basal area) was evaluated, together with the most likely cause of death, such as fire, logging, or SBW outbreak.

We combined field observations of SBW‐induced tree mortality and annual aerial defoliation estimates from aerial surveys conducted by the Government of Québec (1967–2000), which were conducted on different spatial scales (i.e., 400 m^2^ and 58 km^2^, respectively), to create an outbreak severity classification at the EOP‐scale using the following four levels: null, low, moderate, and severe (see Appendix S1 for detailed methodology). The null level corresponded to EOPs with null aerial defoliation estimates and no field observations of SBW‐induced tree mortality. The low level comprised EOPs without field observations of SBW‐induced tree mortality, but with at least one year of moderate to severe aerial defoliation. The moderate and severe levels included plots in which SBW induced the mortality of 25%–75% and >75% of stand basal area, respectively, regardless of the number of years of moderate to severe aerial defoliation (Figure S1).

**FIGURE 1:**
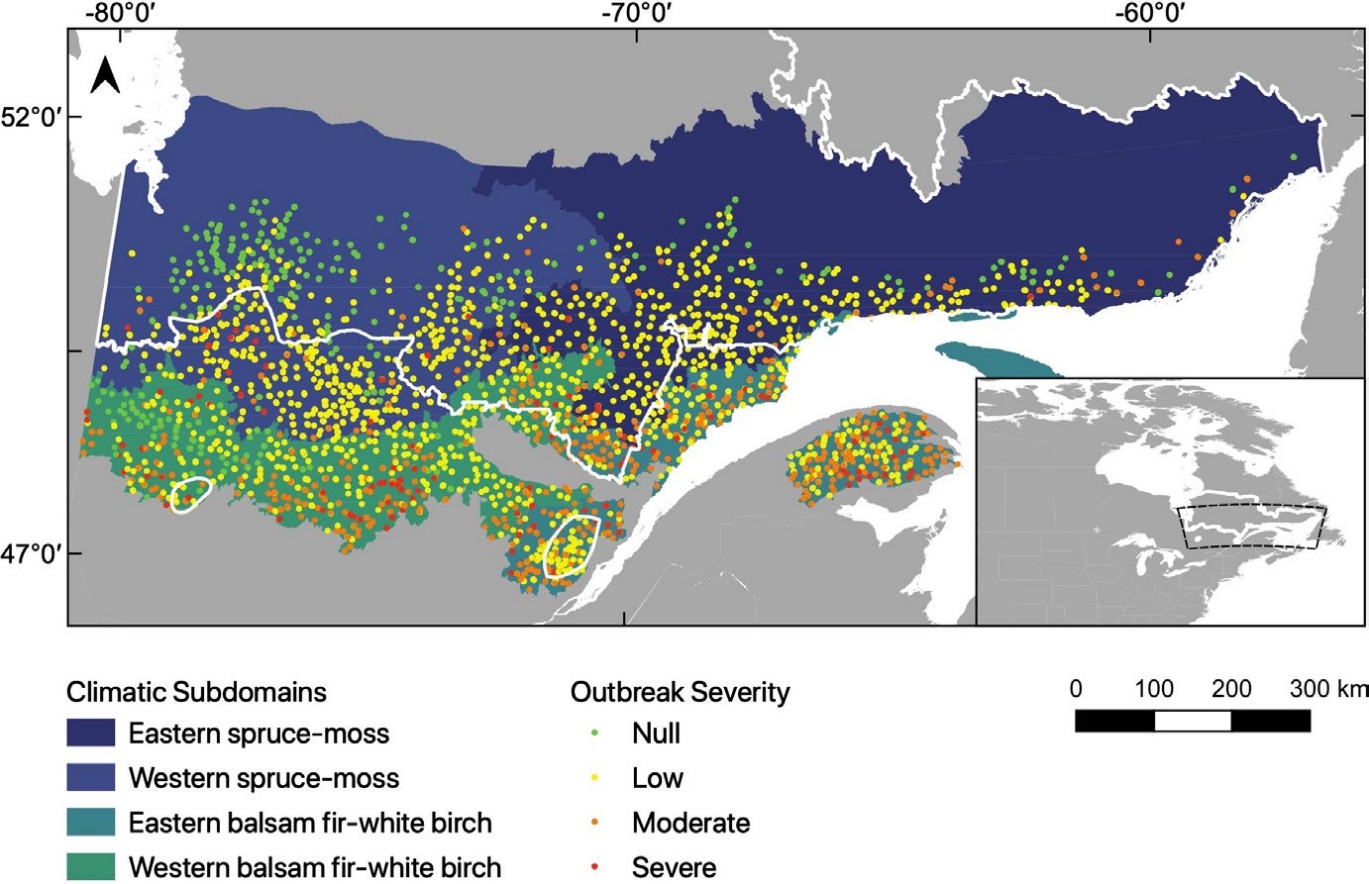
Location of the 6201 plots used to study the effects of spruce budworm (SBW) outbreaks on understory composition in Québec, eastern Canada. Colors represent SBW outbreak severity, ranging from null (no defoliation or mortality) to severe (>75% basal stand area mortality). The white border corresponds to woodland caribou's current distribution in Quebec

To investigate the effect of SBW outbreaks on woodland caribou habitat, we selected all plots located within the balsam fir‐white birch (*Betula papyrifera* Marsh.) and the black spruce (*Picea mariana* Mill. B.S.P.)‐moss bioclimatic domains, which overlap the current woodland caribou distribution or are within its historical range (Courtois et al., 2003). From these plots, we selected those that could be considered preferred woodland caribou habitat because they were as follows: (1) within stands >40 years old (Bastille‐Rousseau et al., 2012); (2) dominated by coniferous trees (>75% of canopy trees; Bastille‐Rousseau et al., 2012; Hins et al., 2009); (3) dominated by balsam fir or spruce (we removed seven and eleven plots dominated by eastern white‐cedar (*Thuja occidentalis* L.) and unknown species, respectively); and (4) unaffected by recent (<40 years) disturbances other than SBW outbreaks, such as fire or logging, which are avoided by caribou (Bastille‐Rousseau et al., 2012; Hins et al., 2009). To this selection, we added all plots corresponding to moderate and severe levels of our outbreak severity classification (>25% SBW‐induced tree mortality). Given that SBW‐induced mortality is strongly correlated with the proportion of pre‐outbreak host tree abundance (Bouchard et al., 2005), it is likely that most of these plots were established in mature stands dominated by conifers prior to the outbreak, thereby constituting potentially suitable habitat for caribou. Our final dataset consisted of 6201 plots (Figure 1; Table 1).

**TABLE 1:** Description of the 6201 plots that were selected for analyses regarding the level of outbreak severity for each bioclimatic subdomain

Bioclimatic subdomain	Sampling (median)	Outbreak severity	Outbreak				Fir (*n*)	Spruce (*n*)	Total (*n*)
			Start (median)	End (median)	Duration (mean ± SD)	Defoliation years (mean ± SD)			
Western spruce‐moss	1991	Null	1977	1978	1.2 ± 0.5	0 ± 0	45	560	2069
		Low	1974	1978	3.2 ± 1.9	2.5 ± 1.7	240	1068	
		Moderate	1974	1979	4.3 ± 2.6	3.2 ± 2.5	86	10	
		Severe	1974	1979	5.9 ± 2.4	4.5 ± 2.3	59	1	
Eastern spruce‐moss	1997	Null	1977	1978	1.2 ± 0.5	0 ± 0	115	52	1452
		Low	1976	1979	3.9 ± 3.1	2.7 ± 1.8	674	414	
		Moderate	1976	1979	5.1 ± 3.9	3.5 ± 2.9	170	4	
		Severe	1975	1979	5.7 ± 3.7	4.5 ± 3.1	173	2	
Western balsam fir‐white birch	1993	Null	1974	1976	1.4 ± 0.8	0 ± 0	21	112	1314
		Low	1974	1980	6.7 ± 4.3	5.1 ± 3.3	207	424	
		Moderate	1972	1985	10.7 ± 3.3	8.1 ± 3.0	361	14	
		Severe	1973	1985	10.4 ± 3.6	8.0 ± 3.4	173	2	
Eastern balsam fir‐white birch	1992	Null	—	—	—	—	0	0	1366
		Low	1974	1986	11.1 ± 4.5	6.9 ± 2.8	445	183	
		Moderate	1974	1986	12.5 ± 3.4	8.6 ± 2.6	547	24	
		Severe	1974	1986	13.0 ± 2.7	9.9 ± 2.1	162	5	
Total									6201

Note

Start and end of the outbreak correspond to the first and last year of the outbreak, respectively, as the first year with a non‐zero defoliation record and the last year with a non‐zero defoliation record that was followed by five consecutive years of null defoliation. Defoliation years refer to the mean number of years of moderate to severe defoliation according to aerial surveys, fir and spruce refer to the number of plots that were identified as pre‐outbreak fir‐ or spruce‐dominated stands, and total corresponds to the total number of plots in each of the climatic subdomains.

For each plot in our final dataset, we extracted understory species cover (as the proportion of the 400 m^2^ plot) from the ground layer to the upper shrub layer (<4 m in height) from the inventory dataset. Species cover was set to the mid‐point of each cover class, for example, 90% for class 81%–100%, 70% for class 61%–80%, and so on. Cover was summed for each of the following vegetation groups: coniferous and deciduous tree seedlings and saplings (<4 m‐high, hereafter referred to as coniferous and deciduous tree regeneration); coniferous and deciduous shrubs; ferns; forbs; fruit‐bearing species; horsetails; terricolous lichens; lycopods and bryophytes (see Table S1 for a detailed species classification). Given that a species used for its fruits could also be used for its foliage, fruit‐bearing species were included in two groups. We also extracted the ecological type of each EOP from the MFFP forest inventory database, which is based upon stand physical characteristics, disturbance dynamics, and potential vegetation in late‐seral conditions (MFFP, 2019). We used this variable as an indicator of pre‐outbreak stand composition and classified each EOP as either a pre‐outbreak balsam fir‐ or spruce‐dominated stand. This allowed us to accurately assess the SBW outbreak severity effect on the understory community by controlling for the confounding effect of pre‐outbreak canopy composition, which may influence understory composition (Fourrier et al., 2015).

### 2.2 Statistical analyses

Using generalized linear models, we investigated the impact of an increasing SBW outbreak severity on understory vegetation using the understory groups described in the previous sections. Generalized linear models were implemented for each of these understory groups, using the level of outbreak severity as an ordinal predictor and the percent cover as response variable. We constructed independent models for each combination of bioclimatic subdomain and ecological type. Indeed, the distribution of the EOPs corresponding to the two ecological types was strongly uneven within each of the outbreak severity levels (Table 1) and led to biased understory responses to outbreak severity when used together in a full model. Specifically, most of the plots characterized by a null or low level of outbreak severity consisted of spruce‐dominated stands, whereas the plots affected by moderate and severe outbreaks mostly consisted of fir‐dominated stands. Therefore, the results of a full model were more representative of a switch from spruce‐ to fir‐dominated stands, rather than associated with outbreak severity. Finally, the vegetation and the climatic conditions underlying the definition of each subdomain would be complex if impossible to integrate in a full model, also justifying the implementation of independent models.

Generalized mixed models were constructed using the package “glmmTMB” (Magnusson et al., 2017) in the R environment (R Core Team, 2018). Models were fitted with either a negative binomial or quasi‐Poisson distribution (Table S2) depending on the distribution that best suited our data. The addition of a zero‐inflation term was sometimes necessary, as some understory groups were sparsely distributed across the plots. Models’ residuals were carefully checked to identify outliers and over‐ or under‐dispersion using diagnostic plots and corresponding tests generated by the package “DHARMa” (Hartig, 2017). Spatial autocorrelation was checked using a permutational test for the Moran's I statistic as implemented by the function “moran.mc” of the package “spdep” (Bivand & Wong, 2018). Evidence of spatial autocorrelation was found in preliminary models when using generalized linear models. This issue was addressed by adding Transect ID as a random effect, as it was an efficient and comprehensible way to account for spatial autocorrelation, whereas the inclusion of spatial coordinates as correlation structure resulted in nonconvergence issues.

We also evaluated the response of targeted species that are recognized as forage for caribou, moose, and bear (Table 3) to an increasing SBW outbreak severity. We investigated the impact of SBW outbreak severity on terricolous lichens from the genus *Cladonia*, upon which woodland caribou feeds throughout the year, and graminoids and forbs species, which form a major part of caribou diet during the snow‐free period (Thompson et al., 2015; see Table 3 for additional references). We also investigated the response of multiple fruit‐bearing species, which benefit moose (Finnegan et al., 2017), black bear (*Ursus americanus*; Brodeur et al., 2008) and coyote (*Canis latrans*; Boisjoly et al., 2010), and a range of deciduous shrubs and tree seedlings species, which are recognized as quality forage for moose (Dussault et al., 2005). For investigating the effect of an increasing SBW outbreak severity on individual species, we used the same methodology as that used for understory groups, but this time using a zero‐inflated negative binomial distribution, which better suited the distribution of individual species.

## 3 RESULTS

**FIGURE 2:**
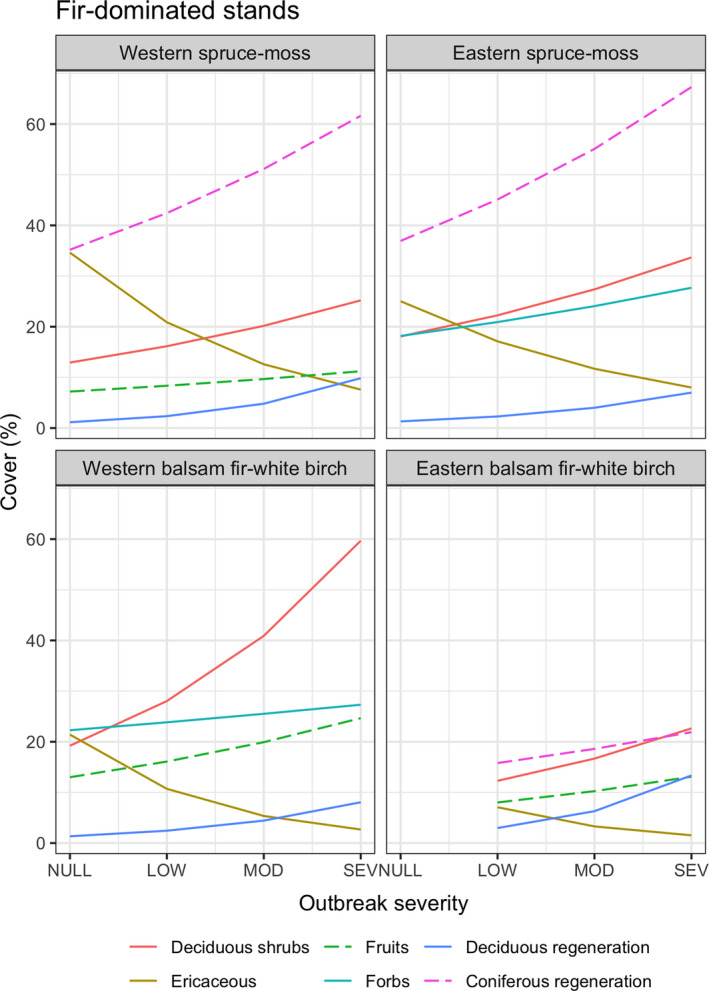
Predicted cover (%) of understory vegetation groups in relation to SBW outbreak severity in pre‐outbreak balsam fir‐dominated stands in the four climatic subdomains. Outbreak severity ranges from null (no defoliation) to severe (>75% of stand basal area killed by SBW). Only understory group significantly (*α* = 0.05) affected by increasing SBW outbreak severity and with changes in cover greater that ~5% between severely affected plots and non‐affected plots are shown

An increasing level of outbreak severity induced significant changes in the abundance of most of the understory groups in the fir‐dominated stands (Figure 2; Table 2). In these stands, our results revealed similar patterns across all four climatic subdomains. An increasing outbreak severity generally benefitted coniferous and deciduous regeneration, deciduous shrubs, fruit‐bearing species, ferns, and forbs. In comparison, the cover of lichens and ericaceous species was negatively related to an increasing outbreak severity. Model predictions indicated that the cover of coniferous regeneration increased by 6 to 30% across all subdomains, with the greatest increases found in the spruce‐moss subdomains (Figure 2). The cover of deciduous regeneration increased by 10%–40%, with a strong increase in the western balsam fir‐white birch subdomain. Models also indicated an increase in the cover of deciduous shrubs, fruit‐bearing species, and forbs of 6%–10%, 4%–12%, and 5%–10%, respectively, with an increasing outbreak severity. The cover of ericaceous species also decreased with an increasing outbreak severity, with decreases ranging between 6% and 27%, and peaking in the western subdomains. A decline in lichen cover was observed and was also more important in the west, but the magnitude of the change in cover was lower than the changes observed for the other understory groups and ranged between 0.5% and 3% across the four subdomains. In contrast with the fir‐dominated stands, the understory of spruce‐dominated stands showed a limited response to increases in SBW outbreak severity. Most the changes in understory group cover were found in the western spruce‐moss subdomain, with limited changes in the other subdomains (Table 2). Changes observed in the western spruce‐moss subdomain were mostly similar to the changes observed in the fir‐dominated stands, except for coniferous regeneration and fruit‐bearing species, which showed opposite trends (Table 2).

**TABLE 2:** Effect of an increasing SBW outbreak severity level on the cover (%) of understory groups (coefficient ± standard error) for fir‐dominated and spruce‐dominated stands in the four climatic subdomains (WS: Western spruce‐moss; ES: eastern spruce‐moss; EF: eastern fir‐white birch; WF: western fir‐white birch)

Understory group	Fir stands				Spruce stands			
	WS	ES	WF	EF	WS	ES	WF	EF
Deciduous regeneration	**0.72 ± 0.08** (9.50)***	**0.56 ± 0.08** (7.20)***	**0.60 ± 0.06** (10.22)***	**0.75 ± 0.05** (14.10)***	**0.58 ± 0.18** (3.21)***	**0.49 ± 0.39** (1.25)	**0.52 ± 0.27** (1.92)	**1.58 ± 0.47** (3.34)***
Coniferous regeneration	**0.19 ± 0.03** (5.63)***	**0.20 ± 0.03** (6.15)***	0.04 ± 0.04 (1.26)	**0.16 ± 0.03** (4.92)***	**−0.16 ± 0.04** (−3.92)***	0.00 ± 0.09 (0.04)	0.03 ± 0.06 (0.45)	−0.22 ± 0.13 (−1.69)
Deciduous shrubs	**0.22 ± 0.06** (3.61)***	**0.21 ± 0.09** (2.18)*	**0.38 ± 0.04** (9.28)***	**0.31 ± 0.05** (6.35)***	0.10 ± 0.08 (1.23)	−0.01 ± 0.17 ( −0.07)	−0.15 ± 0.13 (−1.12)	0.42 ± 0.26 (1.62)
Ericaceous species	**−0.51 ± 0.09** (−5.94)***	**−0.38 ± 0.11** (−3.32)***	**−0.69 ± 0.07** (−10.06)***	**−0.77 ± 0.11** (−6.98)***		0.17 ± 0.14 (1.22)	−0.12 ± 0.08 (−1.51)	−0.51 ± 0.29 (−1.77)
Forbs	0.05 ± 0.04 (1.39)	**0.14 ± 0.04** (3.41)***	**0.07 ± 0.03** (2.15)*	−0.02 ± 0.03 (−0.59)	**0.17 ± 0.06** (2.79)**	0.06 ± 0.12 (0.50)	0.04 ± 0.08 (0.47)	0.14 ± 0.16 (0.86)
Fruit‐bearing species	**0.15 ± 0.05** (2.85)**	0.12 ± 0.06 (1.82)	**0.21 ± 0.04** (5.19)***	**0.25 ± 0.05** (4.53)***	**−0.12 ± 0.06** (−2.03)*	0.12 ± 0.12 (0.95)	−0.05 ± 0.08 (−0.56)	−0.18 ± 0.20 (−0.90)
Graminoids	−0.16 ± 0.15 (−1.10)	0.14 ± 0.18 (0.80)	0.16 ± 0.10 (1.63)	**−0.37 ± 0.13** (−2.90)**	**0.43 ± 0.13** (3.22)**	−0.36 ± 0.39 (−0.92)	**−0.81 ± 0.18** (−4.39)***	−0.45 ± 0.56 (−0.81)
Horsetails	−0.26 ± 0.14 (−1.77)	−0.15 ± 0.45 (−0.33)	**0.26 ± 0.11** (2.33)*			0.11 ± 1.43 (0.08)	−0.19 ± 0.23 (−0.83)	
Ferns	**0.49 ± 0.11** (4.26)***	**0.50 ± 0.12** (4.29)***	**0.35 ± 0.06** (6.25)***	0.03 ± 0.07 (0.41)		−0.49 ± 1.41 (−0.34)	0.62 ± 0.34 (1.79)	
Lichens	**−0.26 ± 0.09** (−2.98)**	**−0.24 ± 0.08** (−3.09)**	**−0.32 ± 0.06** (−5.13)***	**−0.41 ± 0.09** (−4.82)***	**−0.94 ± 0.09** (−10.43)***	0.23 ± 0.22 (1.00)	0.06 ± 1.4 (0.41)	**−0.85 ± 0.41** (−2.09)*

Note

Significant coefficients (α = 0.05) are presented in bold. *Z*‐values associated with each coefficient are shown in parenthesis under the coefficients. The level of significance is also indicated as follows: *means *p* < .05, **means *p* < .01, and ***means *p* < .001. Other cells with values are non‐significant. Empty cells indicate that the model failed to converge, most likely due to a low abundance of the understory group across the plots.

We also investigated the response of individual plant species that were acknowledged as important for caribou, moose, and their predators in the literature (Table 3). The response of these species was coherent with the general response of the understory groups described previously, particularly in fir‐dominated stands. In accordance with our previous results, models indicated that the percent cover of balsam fir and white birch regeneration increased with outbreak severity. In the fir‐dominated stands, the cover of balsam fir regeneration increased by 10%–30% in severely affected plots. White birch was the species associated with the more consistent increase in cover within the deciduous regeneration group, with an increase in cover increase ranging between 4% and 16% in severely affected plots. Deciduous shrubs and fruit‐bearing species such as mountain maple (*Acer spicatum* Lamarck), raspberry (*Rubus idaeus* L.), pin cherry (*Prunus pensylvanica* L.), and mountain‐ash (*Sorbus* spp) also showed a consistent increase with increasing outbreak severity (Table 3). Among these species, the greatest responses were found for raspberry and mountain maple, with increases in cover reaching up to 20% and 32%, respectively, in the balsam fir‐white birch subdomains. In accordance with the general decline in lichen cover associated with more severe SBW outbreaks, lichens form the genus *Cladonia* also consistently decreased with increasing outbreak severity across the study area.

**TABLE 3:** Observed mean cover (%) of the targeted species for caribou conservation for each SBW outbreak severity class and effect of an increasing outbreak severity for fir‐dominated and spruce‐dominated stands in the four climatic subdomains (WS: Western spruce‐moss; ES: eastern spruce‐moss; EF: eastern fir‐white birch; WF: western fir‐white birch)

Species	fr	Mean cover (% (SD))				Fir stands				Spruce stands				Associated animal
		Null	Low	Mod	Sev	WS	ES	EF	WF	WS	ES	EF	WF	
**Bryophytes**														
*Pleurozium schreberi*		36.9 (26.4)	41.1 (24.9)	24.7 (24.2)	19.7 (21.5)	−	−				+			Caribou^12,18,19^
*Sphagnum magellanicum*		1.2 (5.8)	0.8 (4.4)	0.3 (2.6)	0.6 (4.2)		−							Caribou^18,19^
**Coniferous tree regeneration**														
*Abies balsamea*		7.2 (14.2)	11.8 (15.1)	27.9 (22.2)	38.4 (27.5)	+	+	+	+		+		+	Moose^5,8,16^
**Deciduous tree regeneration**														
*Acer rubrum*		<0.1 (0.2)	<0.1 (0.2)	0.5 (2.6)	0.3 (2.0)	+					+			Moose^10^
*Betula papyrifera*		0.6 (2.0)	1.6 (3.2)	5.7 (7.7)	14.2 (14.9)	+	+	+	+	+	+		+	Moose^8,10,16^; Caribou^6^
*Populus tremuloides*		0.1 (0.7)	<0.1 (0.3)	0.2 (0.7)	0.1 (0.3)			+						Moose^10^; Caribou^6^
**Deciduous shrubs**														
*Acer spicatum*		0.1 (1.0)	0.7 (4.1)	11.6 (18.6)	13.6 (21.5)	+	+	+	+	+	+			Moose^5^
*Alnus alnobetula* subsp. *crispa*		2.7 (7.7)	1.3 (4.4)	0.8 (4.2)	0.5 (2.7)		−	−			−		−	Caribou^1,6,12^; Moose^7,12^
*Alnus incana* subsp. *rugosa*		5.5 (13.9)	3.8 (10.8)	2.8 (9.5)	5.6 (13.9)			+				−		Caribou^1,6,12^; Moose^4,7,12^
*Amelanchier* sp.		0.8 (1.9)	1.8 (3.6)	0 (0.0)	1.9 (4.2)	−	−	+			+		+	Caribou^6^; Bear^2,9,11^
*Betula glandulosa*		0.3 (1.4)	0.1 (0.8)	<0.1 (0.1)	<0.1 (0.0)							−	−	Caribou^1,6,12^; Moose^7^
*Betula pumila*		0.1 (0.7)	<0.1 (0.3)	<0.1 (0.0)	<0.1 (0.1)						−			Caribou^1,12^; Moose^7^
*Corylus cornuta*		<0.1 (0.0)	0.1 (1.4)	1.7 (6.7)	1.5 (6.8)		+							Moose^10^; Bear^11^
*Lonicera* spp.		0.1 (1.5)	<0.1 (0.4)	0.1 (0.5)	0.1 (0.9)	−	+					−		Caribou^6^
*Prunus pensylvanica*	fr	<0.1 (0.3)	<0.1 (0.4)	0.3 (1.2)	0.6 (2.4)	+	+		+	+				Moose^10^; Bear^2,9,11,14^
*Ribes* spp.	fr	0.2 (1.0)	0.2 (1.3)	1 (2.7)	1.4 (2.9)				+					Bear^2,9,11,13,14^; Moose^7^
*Rosa acicularis*		<0.1 (0.0)	<0.1 (0.1)	0 (0.0)	0 (0.0)									Caribou^6,12^
*Rubus idaeus*	fr	0.1 (0.9)	0.2 (1.9)	3 (9.6)	7.9 (15.6)	+	+	+	+	+				Moose^10^; Bear^2,3,9,11,13,14^
*Salix* sp.		2.1 (4.1)	1.2 (3.2)	0.1 (0.8)	0.2 (1.1)	−	−		−		−			Moose^10^
*Sorbus* spp.	fr	0.3 (1.2)	0.8 (1.4)	2.2 (3.1)	2.8 (3.6)	+	+	+	+	+	+		+	Bear^9,11,13,14^
*Viburnum edule*	fr	0.1 (0.3)	0.1 (0.8)	0.3 (0.8)	0.4 (1.3)				+					Bear^3,9,11,14^; Caribou^12^; Moose^7^
**Ericaceous species**														
*Empetrum nigrum*	fr	0.1 (0.9)	<0.1 (0.3)	<0.1 (0.1)	<0.1 (0.0)									Bear^9^; Caribou^6,17 (berries)^
*Rhododendron canadense*		0.1 (1.3)	0.1 (1.4)	<0.1 (0.4)	0 (0.0)									Moose^7^
*Rhododendron groenlandicum*		29.3 (24.8)	16.4 (20.1)	1.7 (7.8)	1.6 (5.2)	−	−	−	−	−			−	Moose^7^
*Vaccinium* spp.	fr	9.9 (9.8)	7.6 (9.3)	2 (4.3)	2.2 (5.7)	−	−	−	−		−		−	Bear^2,3,9,14^; Caribou^6,17 (berries)^; Moose^7^
**Forbs**														
*Cornus canadensis*		2.1 (3.3)	3.5 (4.9)	6.1 (8.3)	5.2 (7.2)			+	+				+	Caribou^1,12^; Bear^3,9,11,14^
*Chamaenerion angustifolium subsp. angustifolium*		0.1 (0.5)	0.1 (0.6)	0.1 (0.5)	0.3 (1.8)	+								Caribou^12^; Moose^7^
*Rubus chamaemorus*	fr	1.4 (3.7)	0.7 (2.8)	0.2 (1.8)	<0.1 (0.3)	−							−	Caribou^6,12^
**Graminoids**														
*Carex* sp.		1.5 (5.0)	1.2 (3.7)	0.7 (2.4)	0.8 (2.0)		−				−		+	Caribou ^1,17,19^; Moose^7^; Bear^2,13,14^
**Horsetails**														
*Equisetum* spp.		2.2 (5.5)	0.7 (2.6)	0.3 (1.4)	0.5 (2.0)				−				−	Caribou^1,4,12,19^; Moose^7^
**Lichens**														
*Cladonia* spp.		6.3 (19.7)	2.2 (11.7)	0.8 (1.1)	0.9 (1.2)	−	−	−	−				−	Caribou^6,15,19^

Note

Outbreak severity ranges from null (no defoliation) to severe (>75% of stand basal area killed by SBW). Only significant effects (positive or negative; + or −) of an increasing SBW outbreak severity are reported (*α* = 0.05). The “fr” column indicates that associated species was also considered a fruit‐bearing species. Shaded cells indicate that model failed to converge, most likely due to a too scarce abundance of the understory species across the plots. 1. Bergerud (1972); 2. Boileau et al. (1994); 3. Brodeur et al. (2008); 4. Christopherson (2018); 5. Crête and Jordan (1981); 6. Denryter et al. (2017); 7. Finnegan et al. (2017); 8. Franklin and Harper (2016); 9. Hébert et al. (2008); 10. Lautenschlager et al. (1997); 11. Leblanc (2000); 12. MacDonald et al. (2020); 13. Mosnier, Ouellet, et al. (2008); 14. Romain et al. (2013); 15. Russel et al. (1993); 16. Smith et al. (2010); 17. Thomas et al. (1996); 18. Thompson et al. (2012); 19. Thompson et al. (2015).

## 4 DISCUSSION

Understory development is important for woodland caribou populations, mostly because it benefits moose, which ultimately leads to an increased risk of wolf predation (James et al., 2004; Nadeau Fortin et al., 2016). SBW outbreaks tend to create canopy openings that are more diffuse and less severe than other disturbances such as wildfire or clearcutting (Bouchard et al., 2008). On the other hand, and even if they are less severe locally, these outbreaks cover huge regions and have the potential of generating major impacts on woodland caribou habitats at the regional level (Labadie et al., 2021). We reported a substantial effect of SBW outbreak on the understory of eastern boreal forests of North America, which magnitude increased with outbreak severity. SBW outbreaks may thus reduce habitat quality for animal species that depend on the presence of mature coniferous stands, such as woodland caribou (Hins et al., 2009). In a context where forest harvesting has already fragmented mature coniferous forest habitats (Bastille‐Rousseau et al., 2012; Courtois et al., 2007), SBW disturbances might further decrease the value of the residual patches and their contribution to the conservation of woodland caribou populations. At the regional level, we showed that vegetation response varied among the four climatic subdomains, together with variation in the relative proportion of severely affected plots (Table 1). Variation in the proportion of severely affected plots was likely influenced by differences in the relative abundance of fir, the most vulnerable species, which tends to be higher in warmer (southern) and more humid (eastern) subdomains. The biological performance of the spruce budworm itself may also contribute to the difference in the proportion of severely affected plots, as it tends to cause less damage in colder climatic conditions (Régnière et al., 2012).

### 4.1 Influence of outbreak severity on understory

Not surprisingly, our results highlighted a greater influence of outbreaks in balsam fir‐dominated stands compared to spruce‐dominated stands, which was revealed through both a greater number of severely affected plots and a more consistent response of the understory. Given that balsam fir is more vulnerable to SBW defoliation than spruce (Hennigar et al., 2008), fir‐dominated stands suffered greater tree mortality, which likely increased transmitted light availability (D’Aoust et al., 2004) and altered competitive dynamics in the understory (Kneeshaw & Bergeron, 1998). Accordingly, even though spruce‐ and fir‐dominated stands experienced similar levels of defoliation, the lower mortality of spruce following SBW defoliation resulted in a limited response of the understory layers.

Our results indicated a general increase in deciduous shrubs and tree regeneration cover with increasing outbreak severity in fir‐dominated stands. The relatively high abundance of the coniferous regeneration is coherent with previous findings showing that even if the SBW generates an important mortality in mature trees, seedlings, and saplings tend to persist through outbreaks (Bouchard & Pothier, 2010). A positive response was also observed for deciduous species, particularly in fir‐dominated stands, which was likely promoted by the relatively common presence of several deciduous species in this stand type in pre‐outbreak conditions (Kemball et al., 2005). Following a disturbance, these species may rapidly take advantage of the increased light and proliferate (Kemball et al., 2005; Kneeshaw & Bergeron, 1998; Kneeshaw & Bergeron, 1998) and can lead in the long‐term to a shift from coniferous to mixed or even deciduous stand compositions (Bouchard et al., 2006; Sánchez‐Pinillos et al., 2019). Change in overstory composition from conifer to hardwood could exert a persistent influence on the understory since overstories dominated by deciduous species tend to allow greater light transmission (Fourrier et al., 2015; Messier et al., 1998).

We observed a general decline in *Cladonia* species cover with increasing SBW outbreak severity that was likely caused by the establishment of aggressive shade‐intolerant species. Understory deciduous may rapidly take advantage of canopy openings following disturbance (Kemball et al., 2005) and prevent light from reaching the ground layer, which may be detrimental for lichens (Chagnon & Boudreau, 2019). Similar dynamics have been reported following a mountain pine beetle outbreak in British Columbia, where lichen cover declined whereas vascular species expanded (Cichowski et al., 2008). Moreover, insect outbreaks tend to increase soil nutrient availability (reviewed by Maynard et al., 2014), which promotes the growth of vascular plants that may outcompete lichens (Haughian & Burton, 2015). Our results also indicated a greater abundance of fruit‐bearing species with increasing outbreak severity, especially in fir‐dominated stands, which was mainly driven by *Rubus idaeus*. This species is considered a disturbance specialist that responds quickly to environmental changes and was also associated with severe outbreaks in balsam fir‐white birch mixed stands (Fourrier et al., 2015). In addition to increased cover, fruit production may benefit from the increased light availability following the outbreaks (Moola & Mallik, 1998). It is possible that some fruit‐bearing ericaceous shrubs that are present under spruce canopies, such as *Vaccinium* spp., experience increased fruit productions even if their abundance decreased with an increasing SBW outbreak severity, a phenomenon that could not be assessed with the data at hand.

### 4.2 Implications for woodland caribou

By promoting the presence of early successional species in the understory of mature boreal stands, SBW outbreaks could affect woodland caribou populations by increasing the presence of predators and moose. The abundance of species such as mountain maple, white birch, and balsam fir has increased in severely affected plots in both fir‐ and spruce‐dominated stands in all bioclimatic subdomains and may enhance habitat quality for moose, for which they represent key forage (Franklin & Harper, 2016; Smith et al., 2010). For example, mountain maple was identified as one of the main food sources in the diet of moose in western Québec where it represented >50% of the food consumed (Crête & Jordan, 1981; Joyal, 1976). Moreover, outbreaks promote complex stand structures that combine the characteristics of both old‐growth and regenerating stands (Martin et al., 2019), which may benefit moose by offering both forage and shelter (Dussault et al., 2005). Increased browsing was previously observed in SBW‐defoliated gaps (Franklin & Harper, 2016; Smith et al., 2010), supporting that moose may select defoliated stands over nondisturbed stands for foraging.

Because wolf density generally increases with increasing moose abundance (Bowman et al., 2010; Gagné et al., 2016), SBW outbreaks may intensify predation on caribou. A greater predator density may also be favored by the increase in fruit‐bearing species abundances within moderately and severely defoliated stands. Berries, including raspberry, the fruit‐bearing species that showed the greatest positive response to increasing outbreak severity constitute one of the main food sources for both black bear (Brodeur et al., 2008; Mosnier, Ouellet, et al., 2008) and coyote (Boisjoly et al., 2010). As these species are important predators of juvenile caribou (Lewis et al., 2017; Pinard et al., 2012), increased berry availability following SBW outbreaks may contribute in intensifying predation pressure on caribou, which constitute one of the main factors limiting caribou populations (Bowman et al., 2010; Courtois et al., 2007; Wittmer et al., 2005, 2007).

Overall, SBW outbreaks appear to promote an understory composition that may be favorable for moose and predators and thus unfavorable for caribou. Still, changes in vegetation cover may not be sufficient to induce effective changes in habitat selection, which is a complex and multifactorial process (Leblond et al., 2011). Future studies are needed to evaluate the influence of SBW outbreaks on forage quality and biomass and specifically investigate the impact of SBW outbreaks on habitat selection of caribou and interacting species.

## 5 MANAGEMENT IMPLICATIONS

The distribution range of woodland caribou in North America is known to be affected by multiple disturbances, including fire, forest logging, oil and gas extraction, and mountain pine beetle outbreak. The results of the present study indicate that SBW outbreaks may also induce major changes in the composition of the boreal forest understory, which are likely to be detrimental for woodland caribou. Specifically, early successional species were abundant in stands that experienced SBW‐induced mortality and could promote the presence of caribou predators and their alternate preys, moose. Such changes may result in a direct increased predation risk and habitat loss for caribou, where its habitat selection is strongly influenced by predator avoidance (Hins et al., 2009; Labadie et al., 2021; McGreer et al., 2015).

To our knowledge, there are no obvious forest management practices that could be undertaken to attenuate SBW impacts on caribou populations. Any salvage logging operation to harvest trees damaged by SBW would further decrease the abundance of mature forests at the landscape level (Labadie et al., 2021). By disturbing the soils and established seedlings, logging would likely increase the abundance of pioneer deciduous species compared to stands that were only affected by SBW. Just like logging, silvicultural interventions aimed specifically at controlling deciduous understory species would likely involve the building of roads, a linear feature that is well known to increase predation risks (Courbin et al., 2009; James et al., 2004). The use of herbicides, which could help control the proliferation of deciduous species even without road access if applied with aircrafts, has been banned in public forests in Quebec (Thiffault & Roy, 2011). The only currently available management option could be the application of biological insecticides such as *Bacillus thuringiensis* ssp. *kurstaki* (Btk) by aircraft to reduce mortality in host tree species (Fuentealba et al., 2019; Johns et al., 2019). However, that measure could become costly or difficult to carry out, particularly across vast or remote areas. Overall, we recommend that currently existing minimal habitat requirements for the conservation of woodland caribou populations (c.f. Environment Canada, 2017) should be reviewed to consider the potential impacts of uncontrolled SBW defoliation, which would facilitate the identification of realistic management options.

## AUTHOR CONTRIBUTIONS

**Catherine Chagnon:** Conceptualization (equal); Formal analysis (lead); Methodology (equal); Writing – original draft (lead). **Mathieu Bouchard:** Conceptualization (equal); Methodology (equal); Writing – original draft (supporting). **David Pothier:** Conceptualization (equal); Methodology (equal); Writing – original draft (supporting).

## Supporting information

Appendix S1

## Data Availability

The data that support the findings of this study are available at https://www.foretouverte.gouv.qc.ca/.

## References

[ece38695-bib-0001] Bastille‐Rousseau, G. , Dussault, C. , Couturier, S. , Fortin, D. , St‐Laurent, M.‐H. , Drapeau, P. , Dussault, C. , & Brodeur, V. (2012). Sélection d’habitat du caribou forestier en forêt boréale Québécoise. Ministère du Développement durable, de l’Environnement, de la Faune et des Parcs.

[ece38695-bib-0002] Bergerud, A. T. (1972). Food habits of Newfoundland caribou. Journal of Wildlife Management, 36, 913–923. 10.2307/3799448

[ece38695-bib-0003] Bergerud, A. T. (1974). Decline of caribou in North America following settlement. The Journal of Wildlife Management, 38(4), 757. 10.2307/3800042

[ece38695-bib-0004] Bivand, R. S. , & Wong, D. W. S. (2018). Comparing implementations of global and local indicators of spatial association. Test, 27(3), 716–748. 10.1007/s11749-018-0599-x

[ece38695-bib-0005] Boileau, F. , Crête, M. , & Huot, J. (1994). Food habits of the black bear, *Ursus americanus*, and habitat use in Gaspesie‐Park, eastern Quebec. Canadian Field‐Naturalist, 108, 162–169.

[ece38695-bib-0006] Boisjoly, D. , Ouellet, J.‐P. , & Courtois, R. (2010). Coyote habitat selection and management implications for the Gaspésie Caribou. Journal of Wildlife Management, 74(1), 3–11. 10.2193/2008-149

[ece38695-bib-0007] Bouchard, M. , & Auger, I. (2014). Influence of environmental factors and spatio‐temporal covariates during the initial development of a spruce budworm outbreak. Landscape Ecology, 29(1), 111–126. 10.1007/s10980-013-9966-x

[ece38695-bib-0008] Bouchard, M. , Kneeshaw, D. , & Bergeron, Y. (2005). Mortality and stand renewal patterns following the last spruce budworm outbreak in mixed forests of western Quebec. Forest Ecology and Management, 204, 297–313. 10.1016/j.foreco.2004.09.017

[ece38695-bib-0009] Bouchard, M. , Kneeshaw, D. , & Bergeron, Y. (2006). Forest dynamics after successive spruce budworm outbreaks in mixedwood forests. Ecology, 87(9), 2319–2329. 16995632 10.1890/0012-9658(2006)87[2319:fdassb]2.0.co;2

[ece38695-bib-0010] Bouchard, M. , Kneeshaw, D. , & Bergeron, Y. (2008). Ecosystem management based on large‐scale disturbance pulses: A case study from sub‐boreal forests of western Quebec (Canada). Forest Ecology and Management, 256(10), 1734–1742. 10.1016/j.foreco.2008.05.044

[ece38695-bib-0011] Bouchard, M. , & Pothier, D. (2010). Spatiotemporal variability in tree and stand mortality caused by spruce budworm outbreaks in eastern Quebec. Canadian Journal of Forest Research, 40(1), 86–94. 10.1139/X09-178

[ece38695-bib-0012] Boucher, Y. , & Grondin, P. (2012). Impact of logging and natural stand‐replacing disturbances on high‐elevation boreal landscape dynamics (1950–2005) in eastern Canada. Forest Ecology and Management, 263, 229–239. 10.1016/j.foreco.2011.09.012

[ece38695-bib-0013] Bowman, J. , Ray, J. C. , Magoun, A. J. , Johnson, D. S. , & Dawson, F. N. (2010). Roads, logging, and the large‐mammal community of an eastern Canadian boreal forest. Canadian Journal of Zoology, 88(5), 454–467. 10.1139/Z10-019

[ece38695-bib-0014] Brodeur, V. , Ouellet, J. P. , Courtois, R. , & Fortin, D. (2008). Habitat selection by black bears in an intensively logged boreal forest. Canadian Journal of Zoology, 86(11), 1307–1316. 10.1139/Z08-118

[ece38695-bib-0015] Canada, E. (2017). *Report on the Progress of Recovery Strategy Implementation for the woodland caribou (*Rangifer tarandus caribou*), boreal population in Canada for the period 2012–2017* . Species at risk act recovery strategy series. Environment and Climate. Change Canada, Ottawa: ix +94 pp.

[ece38695-bib-0016] Chagnon, C. , & Boudreau, S. (2019). Shrub canopy induces a decline in lichen abundance and diversity in Nunavik (Québec, Canada). Arctic, Antarctic, and Alpine Research, 51, 521–532. 10.1080/15230430.2019.1688751

[ece38695-bib-0017] Christopherson, V. (2018). Compétition pour les ressources alimentaires entre le caribou et l’orignal en Gaspésie. Université du Québec à Rimouski.

[ece38695-bib-0018] Cichowski, D. , Williston, P. , & Haeussler, S. (2008). The response of caribou terrestrial forage lichens to mountain pine beetles and forest harvesting in the East Ootsa and Entiako areas: Annual Report – 2007/08 – Year 7. A report to Morice‐Lakes Innovative Forest Practices Agreement, Prince George, B.C., the Bulkley Valley Centre for Natural Resources Research and Management, Smithers, B.C., and Ministry of Environment, Prince George, B.C. 46 p.

[ece38695-bib-0019] COSEWIC . (2002). Assessment and update status report on the woodland caribou (98 pp.). Committee on the Status of Endangered Wildlife in Canada.

[ece38695-bib-0020] Courbin, N. , Fortin, D. , Dussault, C. , & Courtois, R. (2009). Landscape management for woodland caribou: the protection of forest blocks influences wolf‐caribou co‐occurrence. Landscape Ecology, 24(10), 1375–1388. 10.1007/s10980-009-9389-x

[ece38695-bib-0021] Courtois, R. , Ouellet, J. P. , Breton, L. , Gingras, A. , & Dussault, C. (2007). Effects of forest disturbance on density, space use, and mortality of woodland caribou. Ecoscience, 14(4), 491–498.

[ece38695-bib-0022] Courtois, R. , Ouellet, J.‐P. , Gingras, A. , Dussault, C. , Breton, L. , & Maltais, J. (2003). Historical changes and current distribution of Caribou, *Rangifer* *tarandus* . Canadian Field‐Naturalist, 117(3), 399–414. 10.22621/cfn.v117i3.742

[ece38695-bib-0023] Crête, M. , & Jordan, P. A. (1981). Régime alimentaire des orignaux du Sud‐Ouest Québécois pour les mois d’avril à octobre. Canadian Field‐Naturalist, 95, 50–56.

[ece38695-bib-0024] D’Aoust, V. , Kneeshaw, D. , & Bergeron, Y. (2004). Characterization of canopy openness before and after a spruce budworm outbreak in the southern boreal forest. Canadian Journal of Forest Research, 34(2), 339–352. 10.1139/x03-278

[ece38695-bib-0025] Denryter, K. A. , Cook, R. C. , Cook, J. G. , & Parker, K. L. (2017). Straight from the caribou’s (*Rangifer tarandus*) mouth: detailed observations of tame caribou reveal new insights into summer‐autumn diets. Canadian Journal of Zoology, 95, 81–94. 10.1139/cjz-2016-0114

[ece38695-bib-0026] Dickie, M. , Serrouya, R. , McNay, R. S. , & Boutin, S. (2016). Faster and farther: wolf movement on linear features and implications for hunting behaviour. Journal of Applied Ecology, 54, 253–263. 10.1111/1365-2664.12732

[ece38695-bib-0027] Dussault, C. , Quellet, J. P. , Courtois, R. , Huot, J. , Breton, L. , & Jolicoeur, H. (2005). Linking moose habitat selection to limiting factors. Ecography, 28(5), 619–628. 10.1111/j.2005.0906-7590.04263.x

[ece38695-bib-0028] Finnegan, L. , Macnearney, D. , & Pigeon, K. E. (2017). Divergent patterns of understory forage growth after seismic line exploration: Implications for caribou habitat restoration. Forest Ecology and Management, 409, 634–652. 10.1016/j.foreco.2017.12.010

[ece38695-bib-0029] Fourrier, A. , Bouchard, M. , & Pothier, D. (2015). Effects of canopy composition and disturbance type on understorey plant assembly in boreal forests. Journal of Vegetation Science, 26(6), 1225–1237. 10.1111/jvs.12323

[ece38695-bib-0030] Franklin, C. M. A. , & Harper, K. A. (2016). Moose browsing, understorey structure and plant species composition across spruce budworm‐induced forest edges. Journal of Vegetation Science, 27(3), 524–534. 10.1111/jvs.12385

[ece38695-bib-0031] Fryxell, J. M. , Avgar, T. , Liu, B. , Baker, J. A. , Rodgers, A. R. , Shuter, J. , Thompson, I. D. , Reid, D. E. B. , Kittle, A. M. , Mosser, A. , Newmaster, S. G. , Nudds, T. D. , Street, G. M. , Brown, G. S. , & Patterson, B. (2020). Anthropogenic disturbance and population viability of woodland caribou in Ontario. Journal of Wildlife Management, 84(4), 636–650. 10.1002/jwmg.21829

[ece38695-bib-0032] Fuentealba, A. , Dupont, A. , Hébert, C. , Berthiaume, R. , Quezada‐García, R. , & Bauce, É. (2019). Comparing the efficacy of various aerial spraying scenarios using *Bacillus thuringiensis* to protect trees from spruce budworm defoliation. Forest Ecology and Management, 432, 1013–1021. 10.1016/j.foreco.2018.10.034

[ece38695-bib-0033] Gagné, C. , Mainguy, J. , & Fortin, D. (2016). The impact of forest harvesting on caribou‐moose‐wolf interactions decreases along a latitudinal gradient. Biological Conservation, 197, 215–222. 10.1016/j.biocon.2016.03.015

[ece38695-bib-0034] Guindon, L. , Bernier, P. Y. , Beaudoin, A. , Pouliot, D. , Villemaire, P. , Hall, R. J. , Latifovic, R. , & St‐Amant, R. (2014). Annual mapping of large forest disturbances across Canada’s forests using 250 m MODIS imagery from 2000 to 2011. Canadian Journal of Forest Research, 44(12), 1545–1554. 10.1139/cjfr-2014-0229

[ece38695-bib-0035] Hartig, F. (2017). DHARMa: residual diagnostics for hierarchical (multi‐level/mixed) regression models. R package version 0.3.3.0. https://CRAN.R‐project.org/package=DHARMa

[ece38695-bib-0036] Haughian, S. R. , & Burton, P. J. (2015). Microhabitat associations of lichens, feathermosses, and vascular plants in a caribou winter range, and their implications for understory development. Botany‐Botanique, 93, 221–231. 10.1139/cjb-2014-0238

[ece38695-bib-0037] Hébert, R. , Samson, C. , & Huot, J. (2008). Factors influencing the abundance of berry plants for black bears, *Ursus americanus*, in Quebec. Canadian Field‐Naturalist, 122, 212–220.

[ece38695-bib-0038] Hennigar, C. R. , MacLean, D. A. , Quiring, D. T. , & Kershaw, J. A. (2008). Differences in spruce budworm defoliation among balsam fir and white, red, and black spruce. Forest Science, 54(2), 158–166. 10.1093/forestscience/54.2.158

[ece38695-bib-0039] Hins, C. , Ouellet, J.‐P. , Dussault, C. , & St‐Laurent, M.‐H. (2009). Habitat selection by forest‐dwelling caribou in managed boreal forest of eastern Canada: Evidence of a landscape configuration effect. Forest Ecology and Management, 257, 636–643. 10.1016/j.foreco.2008.09.049

[ece38695-bib-0040] James, A. R. C. , Boutin, S. , Hebert, D. M. , & Rippin, A. B. (2004). Spatial separation of caribou from moose and its relation to predation by wolves. Journal of Wildlife Management, 68(4), 799–809.

[ece38695-bib-0041] Jardon, Y. , Morin, H. , & Dutilleul, P. (2003). Périodicité et synchronisme des épidémies de la tordeuse des bourgeons de l’épinette au Québec. Canadian Journal of Forest Research, 33, 1947–1961. 10.1139/x03-108

[ece38695-bib-0082] Johns, R. C. , Bowden, J. J. , Carleton, D. R. , Cooke, B. J. , Edwards, S. , Emilson, E. J. S. , James, P. M. A. , Kneeshaw, D. , MacLean, D. A. , Martel, V. , Moise, E. R. D. , Mott, G. D. , Norfolk, C. J. , Owens, E. , Pureswaran, D. S. , Quiring, D. T. , Régnière, J. , Richard, B. , & Stastny, M. (2019). A conceptual framework for the spruce budworm early intervention strategy: Can outbreaks be stopped? Forests, 10(10), 910. 10.3390/f10100910

[ece38695-bib-0042] Joyal, R. (1976). Winter foods of moose in La Vérendrye Park, Québec: an evaluation of two browse survey methods. Canadian Journal of Zoology, 54, 1765–1770. 10.1139/z76-205

[ece38695-bib-0043] Kemball, K. J. , Wang, G. G. , & Dang, Q. L. (2005). Response of understory plant community of boreal mixedwood stands to fire, logging, and spruce budworm outbreak. Canadian Journal of Botany, 83(12), 1550–1560. 10.1139/b05-134

[ece38695-bib-0044] Kneeshaw, D. D. , & Bergeron, Y. (1998). Canopy gap characteristics and tree replacement in the southeastern boreal forest. Ecology, 79(3), 783–794.

[ece38695-bib-0045] Labadie, G. , McLoughlin, P. D. , Hebblewhite, M. , & Fortin, D. (2021). Insect‐mediated apparent competition between mammals in a boreal food web. Proceedings of the National Academy of Sciences, 118(30), e2022892118. 10.1073/pnas.2022892118 PMC8325153 34282006

[ece38695-bib-0046] Lautenschlager, R. A. , Crawford, H. S. , Stokes, M. R. , & Stone, T. L. (1997). Forest disturbance type differentially affects seasonal moose forage. Alces, 33, 49–73.

[ece38695-bib-0047] Leblanc, N. (2000). Sélection d’habitats et utilisation du milieu par l’ours noir (*Ursus americanus*) dans une aire protégée de dimension restreinte: le parc national Forillon. M.Sc., Université Laval.

[ece38695-bib-0048] Leblond, M. , Frair, J. , Fortin, D. , Dussault, C. , Ouellet, J.‐P. , & Courtois, R. (2011). Assessing the influence of resource covariates at multiple spatial scales: an application to forest‐dwelling caribou faced with intensive human activity. Landscape Ecology, 26(10), 1433–1446. 10.1007/s10980-011-9647-6

[ece38695-bib-0049] Lewis, K. P. , Gullage, S. E. , Fifield, D. A. , Jennings, D. H. , & Mahoney, S. P. (2017). Manipulations of black bear and coyote affect caribou calf survival. Journal of Wildlife Management, 81(1), 122–132. 10.1002/jwmg.21174

[ece38695-bib-0050] MacDonald, A. , Bartels, S. F. , Macdonald, S. E. , Pigeon, K. E. , MacNearney, D. , & Finnegan, L. (2020). Wildlife forage cover and composition on pipeline corridors in Alberta: Implications for wildlife conservation. Forest Ecology and Management, 468, 118189. 10.1016/j.foreco.2020.118189

[ece38695-bib-0051] Magnusson, A. , Skaug, H. , Nielsen, A. , Berg, C. , Kristensen, K. , Maechler, M. , Brooks, M. E. (2017). glmmTMB: Generalized linear mixed models using template model builder. R package version 1.0.2.1. https://github.com/glmmTMB/glmmTMB

[ece38695-bib-0052] Martin, M. , Morin, H. , & Fenton, N. J. (2019). Secondary disturbances of low and moderate severity drive the dynamics of eastern Canadian boreal old‐growth forests. Annals of Forest Science, 76(4), 2–16. 10.1007/s13595-019-0891-2

[ece38695-bib-0053] Maynard, D. G. , Paré, D. , Thiffault, E. , Lafleur, B. , Hogg, K. E. , & Kishchuk, B. (2014). How do natural disturbances and human activities affect soils and tree nutrition and growth in the Canadian boreal forest? Environmental Reviews, 22(2), 161–178. 10.1139/er-2013-0057

[ece38695-bib-0054] McGreer, M. T. , Mallon, E. E. , Vander Vennen, L. M. , Wiebe, P. A. , Baker, J. A. , Brown, G. S. , Avgar, T. , Hagens, J. , Kittle, A. M. , Mosser, A. , Street, G. M. , Reid, D. E. B. , Rodgers, A. R. , Shuter, J. , Thompson, I. D. , Turetsky, M. J. , Newmaster, S. G. , Patterson, B. R. , & Fryxell, J. M. (2015). Selection for forage and avoidance of risk by woodland caribou (*Rangifer tarandus caribou*) at coarse and local scales. Ecosphere, 6(112), 1–11. 10.1890/ES15-00174.1

[ece38695-bib-0055] Messier, C. , Parent, S. , & Bergeron, Y. (1998). Effects of overstory and understory vegetation on the understory light environment in mixed boreal forests. Journal of Vegetation Science, 9, 511–520. 10.2307/3237266

[ece38695-bib-0056] MFFP . (2005). Liste des espèces fauniques menacées ou vulnérables au Québec. https://mffp.gouv.qc.ca/la‐faune/especes/especes‐menacees‐vulnerables/

[ece38695-bib-0057] MFFP . (2019). Les guides de reconnaissance des types écologiques. https://mffp.gouv.qc.ca/forets/inventaire/guide‐types‐ecologiques.jsp

[ece38695-bib-0058] Moola, F. M. , & Mallik, A. U. (1998). Morphological plasticity and regeneration strategies of velvet leaf blueberry (*Vaccinium myrtiltoides* Michx.) following canopy disturbance in boreal mixedwood forests. Forest Ecology and Management, 111(1), 35–50. 10.1016/S0378-1127(98)00306-5

[ece38695-bib-0059] Mosnier, A. , Boisjoly, D. , Courtois, R. , & Ouellet, J. P. (2008). Extensive predator space use can limit the efficacy of a control program. The Journal of Wildlife Management, 72(2), 483–491. 10.2193/2006-462

[ece38695-bib-0060] Mosnier, A. , Ouellet, J.‐P. , & Courtois, R. (2008). Black bear adaptation to low productivity in the boreal forest. Ecoscience, 15(4), 485–497. 10.2980/15-4-3100

[ece38695-bib-0061] Nadeau Fortin, M.‐A. , St‐Laurent, M.‐H. , & Sirois, L. (2016). Extensive forest management contributes to maintain suitable habitat characteristics for the endangered Atlantic‐Gaspésie caribou. Canadian Journal of Forestry, 46, 933–942. 10.1139/cjfr-2016-003

[ece38695-bib-0062] Navarro, L. , Morin, H. , Bergeron, Y. , & Girona, M. M. (2018). Changes in spatiotemporal patterns of 20^th^ century spruce budworm outbreaks in eastern Canadian boreal forests. Frontiers in Plant Science, 9, 1905. 10.3389/fpls.2018.01905 30622551 PMC6308396

[ece38695-bib-0083] Peters, W. , Hebblewhite, M. , DeCesare, N. , Cagnacci, F. , & Musiani, M. (2013). Resource separation analysis with moose indicates threats to caribou in human altered landscapes. Ecography, 36(4), 487–498. 10.1111/j.1600-0587.2012.07733.x

[ece38695-bib-0063] Pinard, V. , Dussault, C. , Ouellet, J.‐P. , Fortin, D. , & Courtois, R. (2012). Calving rate, calf survival rate, and habitat selection of forest‐dwelling caribou in a highly managed landscape. Journal of Wildlife Management, 76(1), 189–199. 10.1002/jwmg.217

[ece38695-bib-0064] R Core Team . (2018). R: A language and environment for statistical computing. R Foundation for Statistical Computing.

[ece38695-bib-0065] Régnière, J. , St‐Amant, R. , & Duval, P. (2012). Predicting insect distributions under climate change from physiological responses: Spruce budworm as an example. Biological Invasions, 14(8), 1571–1586. 10.1007/s10530-010-9918-1

[ece38695-bib-0066] Rettie, W. J. , & Messier, F. (2000). Hierarchical habitat selection by woodland caribou: its relationship to limiting factors. Ecography, 23, 466–478. 10.1111/j.1600-0587.2000.tb00303.x

[ece38695-bib-0067] Romain, D. A. , Obbard, M. E. , & Atkinson, J. L. (2013). Temporal variation in food habits of the American black bear (*Ursus americanus*) in the boreal forest of Northern Ontario. Canadian Field‐Naturalist, 127, 118–127. 10.22621/cfn.v127i2.1442

[ece38695-bib-0068] Russel, D. E. , Martell, A. M. , & Nixon, W. A. C. (1993). Range ecology of the Porcupine Caribou herd in Canada. Rangifer, 13(5), 1. 10.7557/2.13.5.1057

[ece38695-bib-0069] Sánchez‐Pinillos, M. , Leduc, A. , Ameztegui, A. , Kneeshaw, D. , Lloret, F. , & Coll, L. (2019). Resistance, resilience or change: Post‐disturbance dynamics of boreal forests after insect outbreaks. Ecosystems, 22(8), 1886–1901. 10.1007/s10021-019-00378-6

[ece38695-bib-0070] Saucier, J.‐P. , Berger, J.‐P. , D’Avignon, H. , & Racine, P. (1994). Le point d’observation écologique, (pp. 1–126). Ministère des ressources naturelles, Direction de la gestion des stocks forestiers.

[ece38695-bib-0071] Smith, C. , Beazley, K. F. , Duinker, P. , & Harper, K. (2010). The impact of moose (*Alces alces andersoni*) on forest regeneration following a severe spruce budworm outbreak in the Cape Breton highlands, Nova Scotia, Canada. Alces: A Journal Devoted to the Biology and Management of Moose, 46, 135–150. https://www.researchgate.net/publication/272819156

[ece38695-bib-0072] Stralberg, D. , Wang, X. , Parisien, M.‐A. , Robinne, F.‐N. , Sólymos, P. , Mahon, C. L. , Nielsen, S. E. , & Bayne, E. M. (2018). Wildfire‐mediated vegetation change in boreal forests of Alberta, Canada. Ecosphere, 9, e02156. 10.1002/ecs2.2156

[ece38695-bib-0073] Sturtevant, B. R. , Cooke, B. J. , Kneeshaw, D. D. , & MacLean, D. (2015). Modeling insect disturbance across forested landscapes: Insights from the spruce budworm. In A. Perera , B. Sturtevant , & L. Buse (Eds.), Simulation modeling of forest landscape disturbances (pp. 93–134). Springer. 10.1007/978-3-319-19809-5_5

[ece38695-bib-0074] Thiffault, N. , & Roy, V. (2011). Living without herbicides in Québec (Canada): Historical context, current strategy, research and challenges in forest vegetation management. European Journal of Forest Research, 130, 117–133. 10.1007/s10342-010-0373-4

[ece38695-bib-0075] Thomas, D. C. , Edmonds, E. J. , & Brown, W. K. (1996). The diet of woodland caribou populations in west‐central Alberta. Rangifer, 16(4), 337. 10.7557/2.16.4.1275

[ece38695-bib-0076] Thompson, I. D. , Bakhtiari, M. , Rodgers, A. R. , Baker, J. A. , Fryxell, J. M. , & Iwachewski, E. (2012). Application of a high‐resolution animal‐borne remote video camera with global positioning for wildlife study: Observations on the secret lives of woodland caribou. Wildlife Society Bulletin, 36(2), 365–370. 10.1002/wsb.130

[ece38695-bib-0077] Thompson, I. D. , Wiebe, P. A. , Mallon, E. , Rodger, A. R. , Fryxell, J. M. , Baker, J. A. , & Reid, D. (2015). Factors influencing the seasonal diet selection by woodland caribou (*Rangifer tarandus tarandus*) in boreal forests in Ontario. Canadian Journal of Zoology, 93(2), 87–98. 10.1139/cjz-2014-0140

[ece38695-bib-0078] Virgin, G. V. J. , & MacLean, D. A. (2017). Five decades of balsam fir stand development after spruce budworm‐related mortality. Forest Ecology and Management, 400, 129–138. 10.1016/j.foreco.2017.05.057

[ece38695-bib-0079] Wittische, J. , Heckbert, S. , James, P. M. A. , Burton, A. C. , & Fisher, J. T. (2021). Community‐level modelling of boreal forest mammal distribution in an oil sands landscape. Science of the Total Environment, 755, 142500. 10.1016/j.scitotenv.2020.142500 33049527

[ece38695-bib-0080] Wittmer, H. U. , McLellan, B. N. , Serrouya, R. , & Apps, C. D. (2007). Changes in landscape composition influence the decline of a threatened woodland caribou population. Journal of Animal Ecology, 76, 568–579. 10.1111/j.1365-2656.2007.01220.x 17439473

[ece38695-bib-0081] Wittmer, H. U. , Sinclair, A. R. E. , & McLellan, B. N. (2005). The role of predation in the decline and extirpation of woodland caribou. Oecologia, 144(2), 257–267. 10.1007/s00442-005-0055-y 15891849

