# Supplementary material for: Impacts of spruce budworm defoliation on the habitat of woodland caribou, moose, and their main predators
Source: Ecol Evol. 2022 Mar 18;12(3):e8695. doi: 10.1002/ece3.8695 (PMC8932078; doi:10.1002/ece3.8695)
Supplement: Supplementary file 1 — Appendix S1 [file ECE3-12-e8695-s001.docx]

**Impacts of spruce budworm defoliation on the habitat of woodland caribou, moose, and their main predators**

**Authors:** Catherine Chagnon^a^, Mathieu Bouchard^ab^ and David Pothier^a^

1. Centre d’étude de la forêt, Département des sciences du bois et de la forêt, Pavillon Abitibi-Price, Université Laval, 2405 rue de la Terrasse, Québec, QC G1V 0A6, Canada
2. Direction de la recherche forestière, Ministère des Forêts, de la Faune et des Parcs, 2700 rue Einstein, Québec, QC G1P 3W8, Canada

Supplementary material

**Detailed methodology for extracting defoliation estimates from historical aerial surveys**

For each EOP, we extracted historical annual SBW defoliations for the period 1967-2000 from aerial surveys conducted by the Ministère des Ressources naturelles (MRN, forerunner of MFFP) of the Government of Québec. Aerial surveys consisted of parallel flight lines that were 4-7 km apart, and which were flown at an elevation of ~240 m; average annual defoliation was estimated along each flight line and reported on a 1:50 000 scale map using a ~58 km^2^ cell grid. Flights were conducted annually between late June in the southwest of the province and August in the northeast, following regional climate and tree phenology (Gray et al., 2000). The following tree defoliation classes were used for subsequent analyses: 0, no observed defoliation; 1, light defoliation (< 35% of annual foliage loss on SBW host species); and 2, moderate to severe defoliation (> 35%) or tree mortality. For each plot, we recorded the first and last year of the outbreak, respectively, as the first year with a non-zero defoliation record and the last year with a non-zero defoliation record that was followed by five consecutive years of null defoliation (Gray et al. 2000; Gray and MacKinnon 2006). We cumulated the number of moderate to severe defoliation years during the outbreak period for each plot to assess the intensity of the SBW outbreak. Light defoliation (< 35%) was not considered in the outbreak intensity measurement because of its limited impact on tree growth (Pothier et al., 2005) and tree mortality (MacLean et al., 2001). We did not consider EOPs that were located in the northernmost part of our study area, since aerial surveys were not conducted in this area, given that it was outside of the SBW distribution range (Gray, 2008).

**
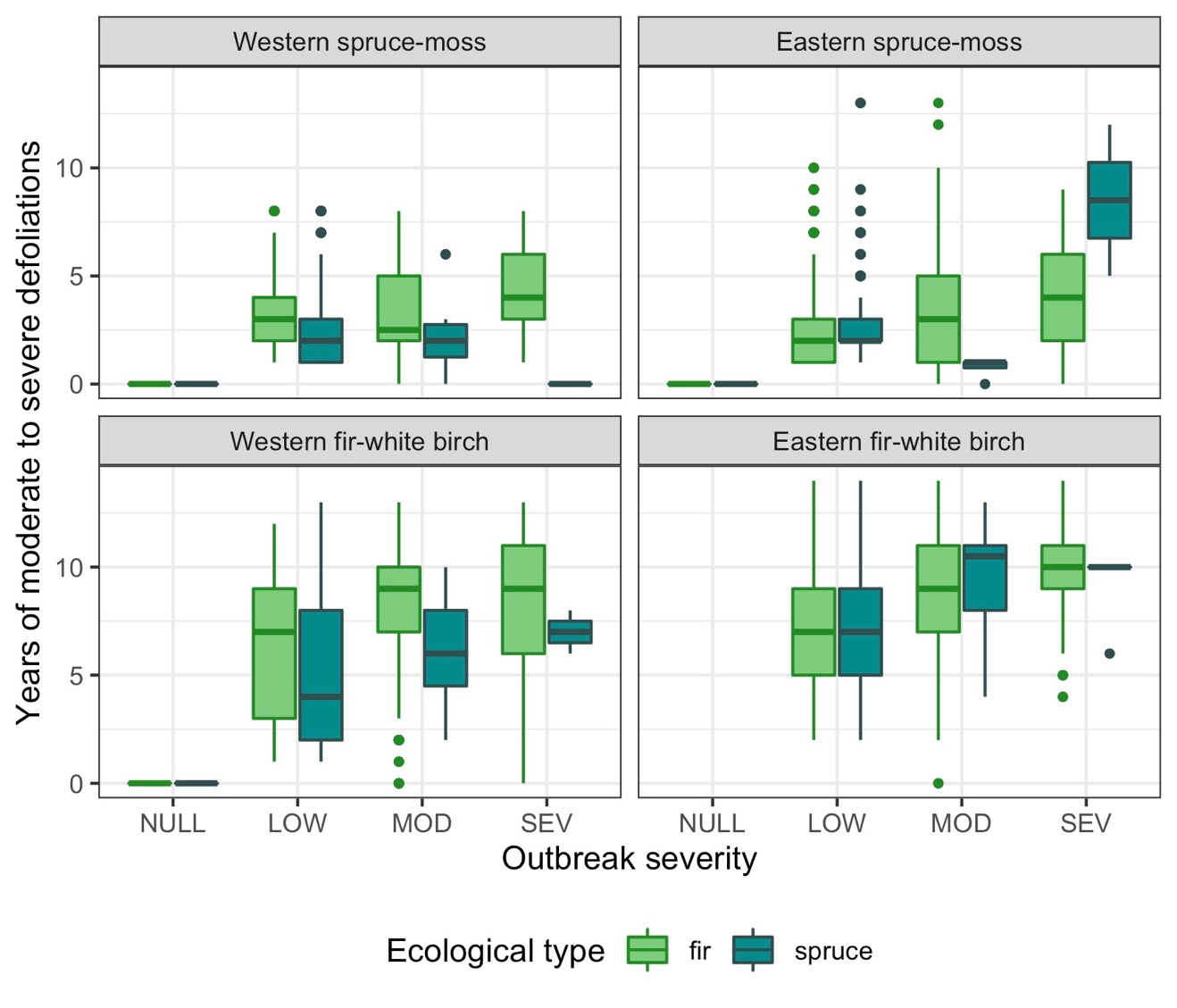
**

**Figure S1.** Number of years of moderate to severe defoliation for each outbreak severity level as assessed by aerial surveys for fir- and spruce-dominated stands in the four climatic subdomains. ﻿Box includes 50% of all values, with the median represented by the line. The whiskers indicate the minimum and maximum values within 1.5 interquartile distances. Outbreak severity ranges from null (no defoliation) to severe (sev; >75% of stand basal area killed by SBW).

**Table S1.** Mean cover (%) of each understory species in each SBW outbreak severity class considering balsam fir- or black spruce-dominated stands (n = 6201 plots). Outbreak severity ranges from null (no defoliation) to severe (>75% of stand basal area killed by SBW). The “fr” column indicates that associated species was also considered a fruit-bearing species.

|  |  | **Cover (%)** | | | |
| --- | --- | --- | --- | --- | --- |
| **Species** | **fr** | **nul** | **low** | **moderate** | **severe** |
| **Bryophytes** |  |  |  |  |  |
| *Pleurozium schreberi* |  | 36.9 | 41.1 | 24.7 | 19.7 |
| *Polytrichum* sp. |  | 0.9 | 0.8 | 0.9 | 0.9 |
| *Ptilidium ciliare* |  | 1.8 | 0.4 | 0.1 | 0.1 |
| *Ptilium crista-castrensis* |  | 7.2 | 9.7 | 3.7 | 2.4 |
| *Rhytidiadelphus triquetrus* |  | 0.1 | <0.1 | 0.1 | <0.1 |
| *Sphagnum fuscum* |  | 5.3 | 2.1 | 0.3 | 0.2 |
| *Sphagnum girgensohnii* |  | 5.1 | 3.5 | 3.6 | 1.3 |
| *Sphagnum magellanicum* |  | 1.2 | 0.8 | 0.3 | 0.6 |
| *Sphagnum* sp. |  | 21.1 | 17.3 | 6.7 | 10 |
| *Sphagnum squarrosum* |  | <0.1 | 0.1 | <0.1 | 0.1 |
| **Coniferous tree regeneration** |  |  |  |  |  |
| *Abies balsamea* |  | 7.2 | 11.8 | 27.9 | 38.4 |
| *Larix laricina* |  | 0.2 | 0.1 | <0.1 | 0.1 |
| *Picea glauca* |  | <0.1 | 0.3 | 1.2 | 0 |
| *Picea mariana* |  | 23.1 | 16.2 | 6.7 | 6.7 |
| *Picea rubens* |  | 0 | <0.1 | <0.1 | <0.1 |
| *Pinus banksiana* |  | <0.1 | <0.1 | <0.1 | 0 |
| *Pinus strobus* |  | 0 | <0.1 | <0.1 | <0.1 |
| *Thuja occidentalis* |  | 0 | <0.1 | 0.3 | 0.1 |
| **Coniferous shrubs** |  |  |  |  |  |
| *Juniperus communis* |  | 0 | <0.1 | 0 | 0 |
| *Juniperus horizontalis* |  | <0.1 | <0.1 | 0 | 0 |
| *Taxus canadensis* |  | <0.1 | 0.1 | 1.2 | 1.1 |
| **Deciduous tree regeneration** |  |  |  |  |  |
| *Acer rubrum* |  | 0 | <0.1 | 0 | 0 |
| *Acer saccharum* |  | <0.1 | <0.1 | 0 | 0 |
| *Betula alleghaniensis* |  | <0.1 | 0.1 | 1.2 | 1.1 |
| *Betula papyrifera* |  | 0.6 | 1.6 | 5.7 | 14.2 |
| *Betula populifolia* |  | 0 | <0.1 | 0 | 0 |
| *Fraxinus nigra* |  | 0 | <0.1 | <0.1 | <0.1 |
| *Ostrya virginiana* |  | <0.1 | <0.1 | <0.1 | 0 |
| *Populus balsamifera* |  | <0.1 | <0.1 | <0.1 | 0 |
| *Populus grandidentata* |  | 0 | <0.1 | 0 | 0 |
| *Populus tremuloides* |  | 0.1 | <0.1 | 0.2 | 0.1 |
| **Deciduous shrubs** |  |  |  |  |  |
| *Acer pensylvanicum* |  | 0 | <0.1 | <0.1 | <0.1 |
| *Acer spicatum* |  | 0.1 | 0.7 | 11.6 | 13.6 |
| *Alnus alnobetula* subsp*. crispa* |  | 2.7 | 1.3 | 0.8 | 0.5 |
| *Alnus incana subsp. rugosa* |  | 5.5 | 3.8 | 2.8 | 5.6 |
| *Amelanchier* sp. |  | 0.8 | 1.8 | 0 | 1.9 |
| *Aronia melanocarpa* |  | <0.1 | <0.1 | 0 | 0 |
| *Betula glandulosa* |  | 0.3 | 0.1 | <0.1 | <0.1 |
| *Betula pumila* |  | 0.1 | <0.1 | <0.1 | <0.1 |
| *Cornus alternifolia* |  | <0.1 | <0.1 | <0.1 | <0.1 |
| *Cornus sericea* | fr | <0.1 | 0.1 | 0.2 | 0.1 |
| *Corylus cornuta* |  | <0.1 | 0.1 | 1.7 | 1.5 |
| *Diervilla lonicera* |  | <0.1 | 0.2 | 1.6 | 1.2 |
| *Endotropis alnifolia* |  | <0.1 | <0.1 | 0 | 0 |
| *Ilex mucronata* |  | 0.4 | 0.7 | 0.5 | 0.5 |
| *Ilex verticillata* | fr | 0 | <0.1 | 0 | 0 |
| *Lonicera canadensis* |  | <0.1 | <0.1 | 0.1 | 0.1 |
| *Lonicera villosa* |  | 0.1 | <0.1 | <0.1 | <0.1 |
| *Myrica gale* |  | <0.1 | <0.1 | 0 | 0 |
| *Prunus pensylvanica* | fr | <0.1 | <0.1 | 0.3 | 0.6 |
| *Prunus virginiana* | fr | <0.1 | <0.1 | <0.1 | <0.1 |
| *Ribes americanum* | fr | 0 | <0.1 | <0.1 | 0 |
| *Ribes cynosbati* | fr | <0.1 | <0.1 | <0.1 | 0 |
| *Ribes glandulosum* | fr | 0.1 | 0.1 | 0.8 | 1.2 |
| *Ribes hirtellum* | fr | 0 | <0.1 | 0 | 0 |
| *Ribes lacustre* | fr | <0.1 | 0.1 | 0.2 | 0.2 |
| *Ribes triste* | fr | <0.1 | 0.1 | 0.1 | 0.1 |
| *Rosa acicularis* |  | <0.1 | <0.1 | 0 | 0 |
| *Rubus idaeus* | fr | 0.1 | 0.2 | 3 | 7.9 |
| *Rubus occidentalis* | fr | 0 | 0 | <0.1 | 0 |
| *Salix* sp. |  | 2.1 | 1.2 | 0.1 | 0.2 |
| *Sambucus canadensis* | fr | 0 | 0 | <0.1 | <0.1 |
| *Sambucus racemosa* | fr | <0.1 | <0.1 | 0.4 | 0.7 |
| *Sorbus americana* | fr | 0.3 | 0.5 | 1.2 | 1.9 |
| *Sorbus decora* | fr | <0.1 | 0.2 | 1 | 0.9 |
| *Spiraea alba* var*. latifolia* |  | 0 | <0.1 | 0 | 0 |
| *Spiraea tomentosa* |  | <0.1 | 0.1 | <0.1 | 0.1 |
| *Viburnum cassinoides* | fr | 0.1 | 0.3 | 1 | 1.2 |
| *Viburnum edule* | fr | 0.1 | 0.1 | 0.3 | 0.4 |
| *Viburnum lantanoides* | fr | <0.1 | <0.1 | 0.2 | <0.1 |
| *Viburnum opulus* var*. americanum* | fr | <0.1 | <0.1 | <0.1 | <0.1 |
| **Ericaceous** |  |  |  |  |  |
| *Andromeda polifolia* var*. latifolia* |  | <0.1 | <0.1 | 0 | 0 |
| *Chamaedaphne calyculata* |  | 3 | 1.1 | <0.1 | <0.1 |
| *Empetrum nigrum* | fr | 0.1 | <0.1 | <0.1 | <0.1 |
| *Kalmia angustifolia* |  | 11.8 | 9.6 | 1.3 | 1.1 |
| *Kalmia polifolia* |  | 0.3 | 0.2 | <0.1 | <0.1 |
| *Rhododendron canadense* |  | 0.1 | 0.1 | <0.1 | 0 |
| *Rhododendron groenlandicum* |  | 29.3 | 16.4 | 1.7 | 1.6 |
| *Vaccinium angustifolium* | fr | 3.4 | 3.5 | 0.8 | 0.7 |
| *Vaccinium cespitosum* | fr | 0.1 | 0.1 | <0.1 | <0.1 |
| *Vaccinium myrtilloides* | fr | 5.8 | 3.7 | 1.2 | 1.5 |
| *Vaccinium uliginosum* | fr | <0.1 | <0.1 | <0.1 | <0.1 |
| **Ferns** |  |  |  |  |  |
| *Athyrium filix-femina* |  | <0.1 | 0.1 | 0.6 | 0.7 |
| *Botrypus virginianus* |  | <0.1 | <0.1 | <0.1 | 0 |
| *Claytosmunda claytoniana* |  | 0.1 | 0.2 | 0.7 | 0.5 |
| *Dennstaedtia punctilobula* |  | 0 | <0.1 | <0.1 | 0 |
| *Dryopteris carthusiana* |  | 0.1 | 0.6 | 3.7 | 2.5 |
| *Dryopteris cristata* |  | 0 | <0.1 | <0.1 | <0.1 |
| *Dryopteris marginalis* |  | <0.1 | <0.1 | <0.1 | 0 |
| *Gymnocarpium disjunctum* |  | 0.1 | 0.2 | 0.5 | 0.4 |
| *Matteuccia struthiopteris* |  | 0 | <0.1 | <0.1 | <0.1 |
| *Onoclea sensibilis* |  | <0.1 | <0.1 | <0.1 | 0 |
| *Osmundastrum cinnamomeum* |  | <0.1 | <0.1 | 0.1 | <0.1 |
| *Parathelypteris noveboracensis* |  | 0 | <0.1 | <0.1 | <0.1 |
| *Phegopteris connectilis* |  | <0.1 | 0.1 | 0.4 | 0.3 |
| *Polypodium virginianum* |  | <0.1 | <0.1 | <0.1 | <0.1 |
| *Polystichum acrostichoides* |  | 0 | 0 | 0 | <0.1 |
| *Polystichum braunii* |  | 0 | <0.1 | 0 | 0 |
| *Pteridium aquilinum* |  | 0.1 | 0.3 | 1.4 | 0.5 |
| **Forbs** |  |  |  |  |  |
| *Maianthemum trifolium* |  | 0.9 | 0.5 | 0.1 | <0.1 |
| *Achillea millefolium* |  | 0 | <0.1 | <0.1 | <0.1 |
| *Actaea pachypoda* |  | 0 | <0.1 | 0 | 0 |
| *Actaea rubra* |  | <0.1 | <0.1 | <0.1 | <0.1 |
| *Actaea* sp*.* |  | 0 | <0.1 | <0.1 | <0.1 |
| *Anaphalis margaritacea* |  | 0 | <0.1 | <0.1 | <0.1 |
| *Anemonastrum canadense* |  | 0 | <0.1 | 0 | 0 |
| *Apocynum androsaemifolium* |  | 0 | <0.1 | <0.1 | <0.1 |
| *Aralia hispida* | fr | 0 | <0.1 | 0 | <0.1 |
| *Aralia nudicaulis* | fr | 0.1 | 0.4 | 2.3 | 1.9 |
| *Arisaema triphyllum* subsp*. triphyllum* |  | 0 | 0 | <0.1 | 0 |
| *Asarum canadense* |  | 0 | <0.1 | 0 | 0 |
| *Aster* sp*.* |  | <0.1 | <0.1 | <0.1 | <0.1 |
| *Caltha palustris* |  | <0.1 | 0 | 0 | 0 |
| *Cardamine diphylla* |  | 0 | <0.1 | <0.1 | 0 |
| *Caulophyllum thalictroides* |  | <0.1 | <0.1 | <0.1 | 0 |
| *Chamaenerion angustifolium¬†subsp.¬†angustifolium* |  | 0.1 | 0.1 | 0.1 | 0.3 |
| *Chimaphila umbellata* |  | 0 | <0.1 | <0.1 | <0.1 |
| *Circaea alpina* |  | <0.1 | <0.1 | 0.1 | <0.1 |
| *Cirsium* sp*.* |  | 0 | <0.1 | <0.1 | 0 |
| *Clintonia borealis* |  | 0.6 | 1.5 | 4.1 | 3 |
| *Coptis trifolia* |  | 0.6 | 0.9 | 1.1 | 1.1 |
| *Corallorhiza maculata* |  | 0 | <0.1 | <0.1 | 0 |
| *Cornus canadensis* |  | 2.1 | 3.5 | 6.1 | 5.2 |
| *Cypripedium acaule* |  | <0.1 | <0.1 | <0.1 | <0.1 |
| *Cypripedium reginae* |  | 0 | <0.1 | 0 | 0 |
| *Dasiphora fruticosa* |  | <0.1 | 0 | 0 | 0 |
| *Dicentra* sp*.* |  | 0 | 0 | <0.1 | 0 |
| *Drosera* sp*.* |  | <0.1 | <0.1 | 0 | 0 |
| *Epigaea repens* |  | 0.1 | 0.1 | <0.1 | <0.1 |
| *Epilobium palustre* |  | 0 | <0.1 | 0 | 0 |
| *Eurybia macrophylla* |  | <0.1 | 0.1 | 0.7 | 0.4 |
| *Eutrochium maculatum* var*. maculatum* |  | 0 | <0.1 | 0 | 0 |
| *Fallopia cilinodis* |  | 0 | 0 | <0.1 | <0.1 |
| *Fragaria* sp*.* | fr | <0.1 | <0.1 | <0.1 | <0.1 |
| *Galium labradoricum* |  | 0 | <0.1 | 0 | 0 |
| *Galium* sp*.* |  | <0.1 | <0.1 | 0.1 | 0.1 |
| *Galium triflorum* |  | <0.1 | <0.1 | <0.1 | <0.1 |
| *Gaultheria hispidula* |  | 4.7 | 3.9 | 2.8 | 2.4 |
| *Gaultheria hispidula* |  | 4.7 | 3.9 | 2.8 | 2.4 |
| *Geocaulon lividum* |  | 0.1 | 0.1 | <0.1 | <0.1 |
| *Geum macrophyllum* |  | <0.1 | <0.1 | <0.1 | 0 |
| *Geum rivale* |  | <0.1 | <0.1 | <0.1 | 0 |
| *Goodyera repens* |  | <0.1 | <0.1 | <0.1 | <0.1 |
| *Goodyera* sp*.* |  | <0.1 | <0.1 | <0.1 | 0 |
| *Habenaria* sp*.* |  | <0.1 | <0.1 | <0.1 | <0.1 |
| *Heracleum maximum* |  | 0 | <0.1 | <0.1 | 0 |
| *Hieracium* sp*.* |  | <0.1 | <0.1 | <0.1 | <0.1 |
| *Hypopitys monotropa* |  | <0.1 | <0.1 | <0.1 | 0 |
| *Impatiens capensis* |  | 0 | <0.1 | <0.1 | <0.1 |
| *Impatiens* sp. |  | 0 | 0 | 0 | <0.1 |
| *Linnaea borealis* |  | 0.4 | 0.7 | 1.7 | 1.4 |
| *Lycopus uniflorus* |  | 0 | <0.1 | <0.1 | 0 |
| *Lysimachia borealis* |  | 0.2 | 0.4 | 1.4 | 0.9 |
| *Maianthemum canadense* |  | 0.5 | 1.1 | 2 | 1.5 |
| *Maianthemum racemosum* |  | 0 | <0.1 | <0.1 | <0.1 |
| *Maianthemum stellatum* |  | 0 | 0 | <0.1 | 0 |
| *Melampyrum lineare* |  | <0.1 | <0.1 | <0.1 | 0 |
| *Mertensia paniculata* |  | 0 | <0.1 | 0 | 0 |
| *Mitella nuda* |  | 0.1 | 0.1 | 0.2 | 0.1 |
| *Moneses uniflora* |  | <0.1 | <0.1 | <0.1 | <0.1 |
| *Monotropa uniflora* |  | <0.1 | <0.1 | <0.1 | <0.1 |
| *Nabalus* sp. |  | <0.1 | <0.1 | <0.1 | <0.1 |
| *Neottia cordata* |  | 0.1 | 0.1 | <0.1 | <0.1 |
| *Oclemena acuminata* |  | <0.1 | <0.1 | 0.5 | 0.3 |
| *Orthilia secunda* |  | <0.1 | 0.1 | 0.1 | <0.1 |
| *Osmorhiza claytonii* |  | 0 | <0.1 | 0 | 0 |
| *Oxalis montana* |  | 0.1 | 1.1 | 6.2 | 3.1 |
| *Petasites frigidus* var*. palmatus* |  | 0.3 | 0.1 | <0.1 | <0.1 |
| *Platanthera orbiculata* |  | <0.1 | <0.1 | <0.1 | <0.1 |
| *Polygonatum pubescens* |  | 0 | <0.1 | <0.1 | 0 |
| *Polygonum* sp*.* |  | 0 | 0 | <0.1 | <0.1 |
| *Potentilla norvegica* |  | 0 | <0.1 | 0 | 0 |
| *Pyrola asarifolia* |  | <0.1 | <0.1 | <0.1 | <0.1 |
| *Pyrola elliptica* |  | <0.1 | <0.1 | <0.1 | <0.1 |
| *Pyrola* sp*.* |  | <0.1 | 0.1 | <0.1 | <0.1 |
| *Ranunculus abortivus* |  | <0.1 | 0 | 0 | 0 |
| *Ranunculus acris* |  | 0 | 0 | <0.1 | 0 |
| *Ranunculus* sp*.* |  | <0.1 | <0.1 | 0 | 0 |
| *Rubus chamaemorus* | fr | 1.4 | 0.7 | 0.2 | <0.1 |
| *Rubus pubescens* | fr | 0.1 | 0.2 | 0.6 | 0.4 |
| *Rubus repens* | fr | 0 | <0.1 | <0.1 | <0.1 |
| *Sarracenia purpurea* |  | <0.1 | <0.1 | 0.4 | 0.7 |
| *Scutellaria galericulata* var*. pubescens* |  | 0 | <0.1 | 0 | 0 |
| *Senecio* sp*.* |  | <0.1 | <0.1 | 0 | 0 |
| *Solidago flexicaulis* |  | <0.1 | <0.1 | <0.1 | <0.1 |
| *Solidago macrophylla* |  | <0.1 | 0.1 | 0.4 | 0.3 |
| *Solidago rugosa* |  | <0.1 | <0.1 | <0.1 | 0 |
| *Solidago* sp*.* |  | <0.1 | <0.1 | <0.1 | <0.1 |
| *Sonchus* sp*.* |  | <0.1 | <0.1 | 0 | 0 |
| *Stellaria* sp*.* |  | 0 | <0.1 | <0.1 | 0 |
| *Streptopus amplexifolius* |  | <0.1 | <0.1 | 0.1 | <0.1 |
| *Streptopus lanceolatus* var*. lanceolatus* |  | <0.1 | 0.1 | 0.3 | 0.2 |
| *Symphyotrichum puniceum* var. *puniceum* |  | <0.1 | <0.1 | <0.1 | 0 |
| *Taraxacum officinale* |  | 0 | <0.1 | <0.1 | <0.1 |
| *Thalictrum dioicum* |  | 0 | <0.1 | <0.1 | <0.1 |
| *Thalictrum pubescens* |  | <0.1 | <0.1 | <0.1 | 0.1 |
| *Tiarella cordifolia* |  | 0 | <0.1 | <0.1 | 0 |
| *Trillidium undulatum* |  | <0.1 | <0.1 | <0.1 | <0.1 |
| *Trillium cernuum* |  | 0 | 0 | <0.1 | <0.1 |
| *Trillium erectum* |  | 0 | <0.1 | <0.1 | <0.1 |
| *Trillium grandiflorum* |  | 0 | <0.1 | 0 | <0.1 |
| *Typha latifolia* |  | <0.1 | 0 | 0 | 0 |
| *Uvularia grandiflora* |  | 0 | <0.1 | 0 | 0 |
| *Vaccinium oxycoccos* |  | 0.5 | 0.1 | <0.1 | <0.1 |
| *Vaccinium vitis-idaea* |  | 0.2 | 0.1 | <0.1 | <0.1 |
| *Vicia cracca* |  | 0 | 0 | <0.1 | 0 |
| *Viola macloskeyi* |  | 0 | <0.1 | 0 | <0.1 |
| *Viola pubescens* var*. pubescens* |  | <0.1 | 0 | 0 | 0 |
| *Viola* sp*.* |  | 0.1 | 0.1 | 0.5 | 0.5 |
| **Graminoids** |  |  |  |  |  |
| *Carex* sp*.* |  | 1.5 | 1.2 | 0.7 | 0.8 |
| *Eriophorum* sp*.* |  | 0 | <0.1 | <0.1 | 0 |
| Other graminoids |  | 0.4 | 0.3 | 0.3 | 0.6 |
| **Horsetails** |  |  |  |  |  |
| *Equisetum* sp*.* |  | 0.6 | 0.5 | 0.2 | 0.4 |
| *Equisetum sylvaticum* |  | 1.6 | 0.2 | <0.1 | 0.1 |
| **Lichens** |  |  |  |  |  |
| *Cladonia mitis* |  | 1.1 | 0.6 | 0.2 | 0.1 |
| *Cladonia rangiferina* |  | 6.3 | 2.2 | 0.4 | 0.3 |
| *Cladonia* sp*.* |  | <0.1 | <0.1 | <0.1 | 0.1 |
| *Cladonia stellaris* |  | 5.6 | 2.1 | <0.1 | <0.1 |
| *Stereocaulon* sp. |  | 0 | <0.1 | <0.1 | 0 |
| **Lycopods** |  |  |  |  |  |
| *Diphasiastrum digitatum* |  | 0 | <0.1 | <0.1 | 0 |
| *Huperzia lucidula* |  | <0.1 | <0.1 | 0.5 | 0.2 |
| *Lycopodium clavatum* |  | <0.1 | <0.1 | <0.1 | 0.1 |
| *Lycopodium complanatum* |  | <0.1 | <0.1 | <0.1 | <0.1 |
| *Lycopodium obscurum* |  | <0.1 | 0.1 | 0.3 | 0.3 |
| *Lycopodium tristachyum* |  | 0 | <0.1 | 0 | 0 |
| *Spinulum annotinum* subsp*. annotinum* |  | 0.7 | 0.6 | 0.8 | 1.4 |

**Table S2.** Distribution (NB: negative binomial; QP: quasi-Poisson, ziNB: zero-inflated negative binomial; ziQP: zero-inflated quasi-Poisson) used in the generalized linear models to investigate the effect of an increasing SBW outbreak severity on understory groups for fir-dominated and spruce-dominated stands in the four climatic subdomains (WS: Western spruce-moss; ES: eastern spruce-moss; EF: eastern fir-white birch; WF: western fir-white birch). Shaded cells indicate that model failed to converge, most likely due to a too scarce abundance of the understory group across the plots. Modelling of bryophyte cover failed across all subdomains.

|  | **Fir stands** | | | | **Spruce stands** | | |  |
| --- | --- | --- | --- | --- | --- | --- | --- | --- |
| **Understory group** | WS | ES | WF | EF | WS | ES | WF | EF |
| Deciduous regeneration | NB | NB | NB | NB | NB | NB | NB | NB |
| Coniferous regeneration | QP | QP | QP | QP | NB | QP | NB | QP |
| Deciduous shrubs | QP | QP | QP | QP | QP | QP | QP | QP |
| Ericaceous | NB | NB | QP | QP |  | NB | QP | QP |
| Forbs | NB | NB | NB | QP | NB | NB | NB | QP |
| Fruit-bearing species | NB | NB | NB | NB | ziNB | QP | QP | NB |
| Graminoids | QP | QP | QP | QP | QP | QP | QP | NB |
| Horsetails | QP | NB | ziQP |  |  | NB | ziNB |  |
| Ferns | QP | QP | ziNB | ziQP |  | NB | QP |  |
| Lichens | NB | NB | QP | QP | NB | NB | NB | NB |

**References**

Gray, D.R. (2008) The relationship between climate and outbreak characteristics of the spruce budworm in eastern Canada. Climatic Change, 87(3–4), 361–383. https://doi.org/10.1007/s10584-007-9317-5

Gray, D.R., Régnière, J., Boulet, B. (2000) Analysis and use of historical patterns of spruce budworm defoliation to forecast outbreak patterns in Quebec. Forest Ecology and Management, 127(1–3), 217–231. https://doi.org/10.1016/S0378-1127(99)00134-6

Gray, D.R., MacKinnon, W.E. (2006) Outbreak patterns of the spruce budworm and their impacts in Canada. Forestry Chronicle, 82(4), 550–561. https://doi.org/10.5558/tfc82550-4

MacLean, D.A., Erdle, T., MacKinnon, W.E., Porter, K., Beaton, K., Cormier, G., Morehouse, S., Budd, M. (2001) The Spruce Budworm Decision Support System: forest protection planning to sustain long-term wood supply. Canadian Journal of Forest Research, 31(10), 1742–1757. <https://doi.org/10.1139/cjfr-31-10-1742>

Pothier, D., Mailly, D., Tremblay, S. (2005) Predicting balsam fir growth reduction caused by spruce budworm using large-scale historical records of defoliation. Annals of Forest Science, 62(3), 261–267. https://doi.org/10.1051/forest:2005018
